# CMOS Image Sensors in Surveillance System Applications

**DOI:** 10.3390/s21020488

**Published:** 2021-01-12

**Authors:** Susrutha Babu Sukhavasi, Suparshya Babu Sukhavasi, Khaled Elleithy, Shakour Abuzneid, Abdelrahman Elleithy

**Affiliations:** 1 Department of Computer Science and Engineering, University of Bridgeport, Bridgeport, CT 06604, USA; ssukhava@my.bridgeport.edu (S.B.S.); susukhav@my.bridgeport.edu (S.B.S.); abuzneid@bridgeport.edu (S.A.); 2 Department of Computer Science, William Paterson University, Wayne, NJ 07470, USA; elleithya@wpunj.edu

**Keywords:** CMOS image sensor, surveillance systems, resolution, dynamic range, frame rate, signal-to-noise ratio

## Abstract

Recent technology advances in CMOS image sensors (CIS) enable their utilization in the most demanding of surveillance fields, especially visual surveillance and intrusion detection in intelligent surveillance systems, aerial surveillance in war zones, Earth environmental surveillance by satellites in space monitoring, agricultural monitoring using wireless sensor networks and internet of things and driver assistance in automotive fields. This paper presents an overview of CMOS image sensor-based surveillance applications over the last decade by tabulating the design characteristics related to image quality such as resolution, frame rate, dynamic range, signal-to-noise ratio, and also processing technology. Different models of CMOS image sensors used in all applications have been surveyed and tabulated for every year and application.

## 1. Introduction

Nowadays, humankind is more dependent on technology, especially in automotive, military, space, wireless sensor networks, and the internet of things for surveillance purposes. To make life easier, these fields have used a lot of convenient methods. We need to think of imaging technology for emerging imaging systems in all the mentioned applications over the past decade. The improvements and advancements are still going on to miniaturize these applications with high speed and high performance for incorporation in a micro area. Due to their amazing performance advantages over CCDs, CMOS image sensors (CIS) have grabbed huge attention in most applications from the past decade. To explain CIS’s significance, we herein review the literature since 2009 in which applications were developed using CIS.

CIS is implemented in applications like HODET [1] in intelligent surveillance systems (ISS), Fish Eye [2] in the automotive field, KINECT-KII [3] in the internet of things (IoT), CUBESAT and MENZ SAT [4] in space, IPASS [5] in the military, TIGERCENCE [6] in wireless sensor networks (WSN), etc.

**Figure 1:**
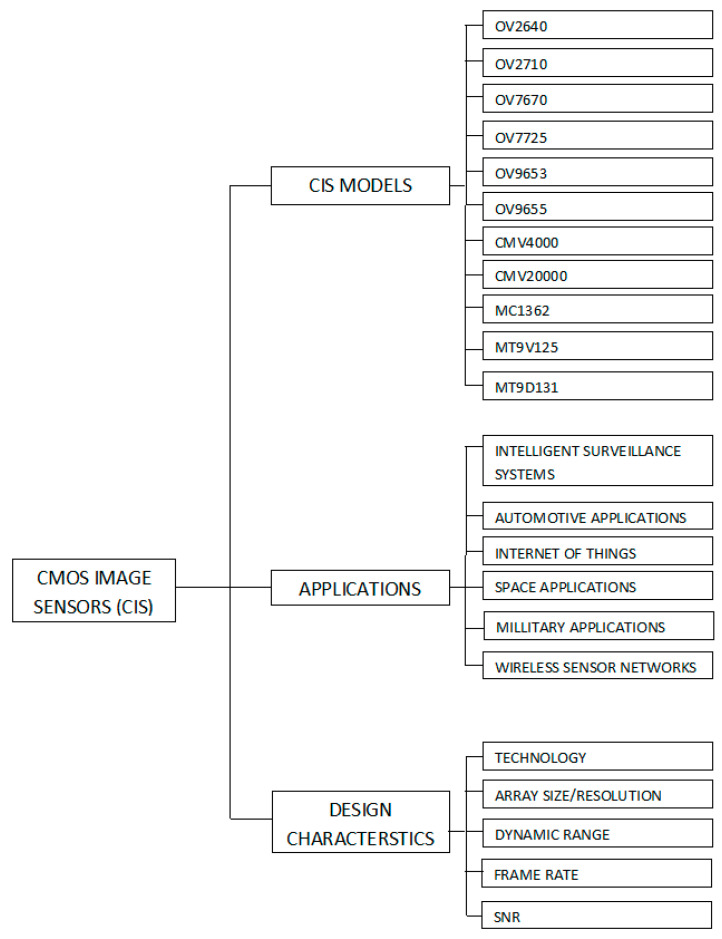
Taxonomy of CMOS image sensor applications.

In our survey, we concentrate on literature related to the types and applications of CIS in various demanding areas. Our contributions are listed below:

- We have conducted the first state-of-the-art comprehensive survey on CIS from an applications’ perspective in different predominant fields, which was not done before.
- A novel taxonomy has been introduced by us in which work is classified in terms of CIS models, applications, and design characteristics, as shown in Figure 1 and Appendix A Table A1.
- We have noted the limitations and future directions and related works are highlighted.

A novel taxonomy and CMOS image sensor types are discussed in Section 2. In Section 3, CMOS image sensor applications are classified with respect to their different application fields. The design characteristics of CMOS image sensors are explained, and corresponding functioning parameters are tabulated in Section 4. Limitations are discussed in Section 5. In Section 6, conclusions are offered.

## 2. Taxonomy and Related Work

We discuss the CMOS image sensor, types of CMOS image sensors, and advantages of CIS over CCD in this section.

### 2.1. CMOS Image Sensors and Their Types

**Figure 2:**
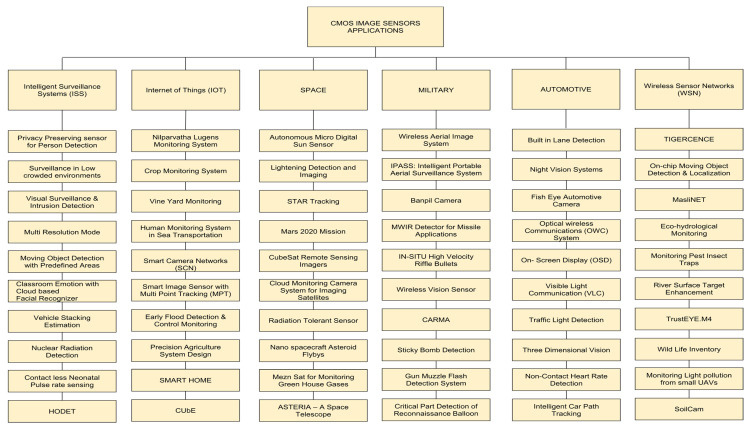
Classification of CMOS image sensor based applications in various fields for surveillance.

In this section, we discuss the basic CMOS image [7] sensor and its working principle with its applications in surveillance systems is shown in Figure 2. Later the types of the CMOS image sensors are addressed. Finally, the advantages of CIS over CCD are discussed clearly (show in Table A2).

#### 2.1.1. CMOS Image Sensor

**Figure 3:**
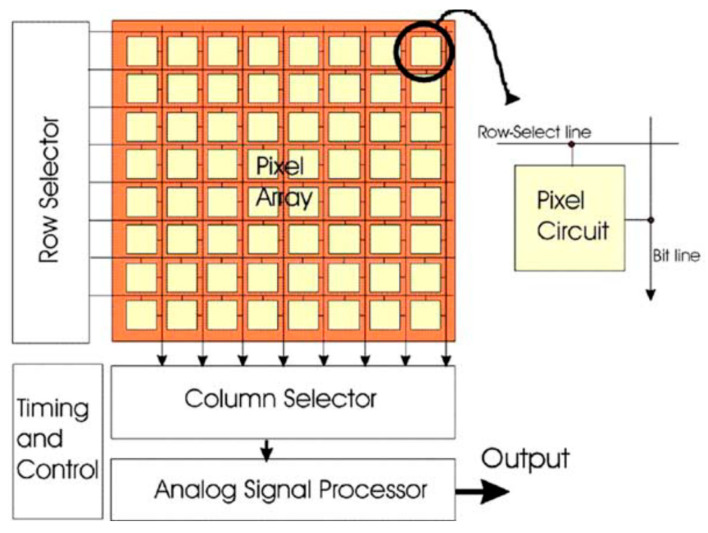
CMOS image sensor architecture. Adapted with from [8] with permission of Elsevier, 2006.

An image sensor is a sensor that converts incident light photons into electrons. CMOS image sensors contain an imaging area, including an array of pixels, readout circuitry and horizontal and vertical access circuitry. The CMOS image sensor architecture is shown in Figure 3 below.

Some traditional imagers are used, namely photodiodes, photogates, and charge coupled devices. Photodiodes convert the incident light into electrons or an electrical current. The current will be produced when photons are extracted into the photodiode. Nowadays, CMOS image sensors use photodiode pixels. Secondly, photogates need to operate at high voltages to collect the electrons generated by incident photons. Thirdly, the charge coupled device, also called CCD, and has architectures involving a combination of parallel and series connection of capacitors. With external circuitry, every capacitor will transfer its collected electric charge to the next capacitor.

#### 2.1.2. Pixel Structures

The pixel structures are two types that were developed earlier in which they created passive pixel sensors (PPS) primarily, and later to increase the quality of an image, they developed an active pixel sensor (APS). PPS and APS’s main difference is PPS consists of one transistor in a pixel, whereas APS started with three transistors in a pixel, and later they increased to four transistors in a pixel.

##### Passive Pixel Sensors

**Figure 4:**
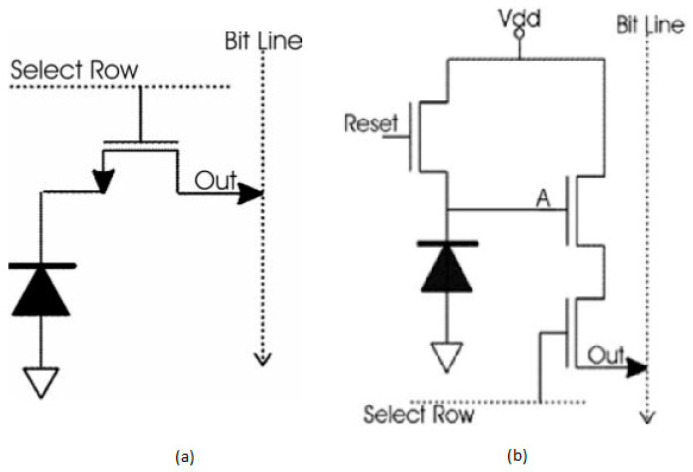
(**a**) Passive pixel sensor; (**b**) Active pixel sensor. Adapted with permission from [8], Elsevier, 2006.

These are the very first pixel structures used in CMOS image sensors. The circuitry consists of photodiodes, but there is no amplification inside the structure. In these pixel structures, each pixel has a photodiode and a transistor and gets connected to a readout structure. Once the pixel addressing will be done by opening the select row transistor, the pixel will be reset with the bit line and the select row transistor, as shown in Figure 4a.

Due to the considerable column’s capacitance, high noise and low sensitivity will occur in passive pixel sensors. Because of this phenomenon, the PPS lagged behind the active pixel sensors and made the active pixel sensors to lead the pixel circuits [8].

##### Active Pixel Sensors

In this type of sensor, an amplifier is incorporated into the pixel to increase the pixel performance. This amplifier is nothing but a source follower, which is inactive during the state of readout only, as shown in Figure 4b. The dissipation of power is low compared to the conventional CCD’s.

A few drawbacks are also present in the APS, which includes high fixed pattern noise, which occurs because of changes in the wafer process that lead to variations in the transistor’s threshold level and gain. The remedy to reduce this fixed pattern noise (FPN) is the introduction of a sampling circuit named correlated double sampling (CDS) circuit. This CDS circuit ultimately reduces the video background offsets caused by the variations in the threshold level [9].

#### 2.1.3. CIS Types

**Figure 5:**
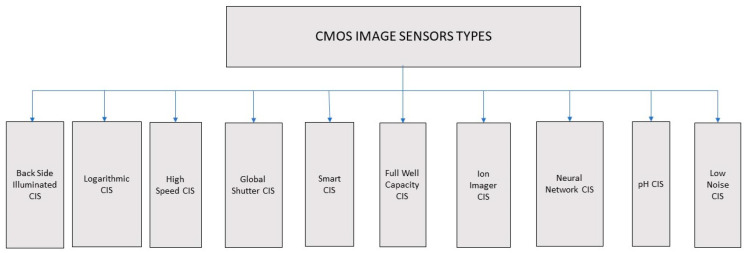
Types of CMOS image sensors.

Different types of CMOS image sensors have evolved to overcome the drawbacks and meet the application requirements. Some of the types of CMOS image sensors are backside illuminated CIS, logarithmic CIS, high-speed CIS, global shutter CIS, smart CIS, full well capacity CIS, ion image sensor CIS, neural network CIS, pH CIS, low noise CIS are shown in Figure 5.

#### 2.1.4. Advantages of CMOS Image Sensors over Charge Coupled Devices

- Generally, CCD sensors require specific fabrication, a dedicated and expensive manufacturing process. In contrast, CMOS image sensors are made using standard manufacturing facilities and can be made at a very low cost.
- The pixel architectures, i.e., APS, usually consume significantly less power, which is a hundred times less than CCD sensors. This parameter makes CMOS image sensors build compact applications that depend on batteries such as cell phones, laptops, etc. However, CCD applications consume immense power due to capacitive devices requiring more clock swings and control signals externally. And also, to operate, CCD systems require voltage regulators with additional power supplies a lot.
- Due to faster frame rates, CMOS APS architectures have been selective imager component in machine vision and motion estimation applications compared to PPS CMOS and CCDs.
- The other advantage of CMOS over CCD is its high integrability on the chip, which allows digital signal processing functions like image stabilization, image compression, multi-resolution imaging, wireless control, color encoding, etc., to call CIS as smart CIS than CCD.

## 3. CMOS Image Sensor Applications

**Figure 6:**
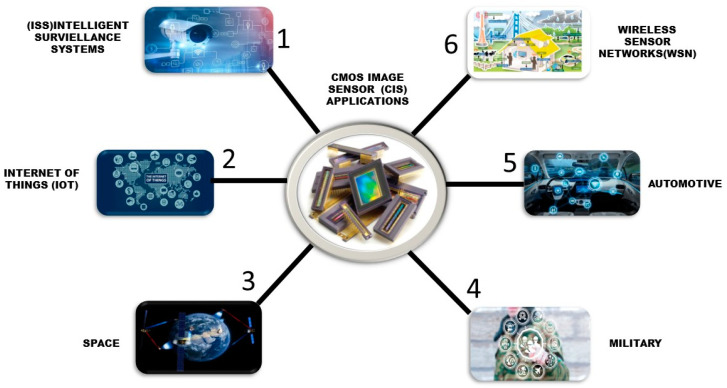
Applications of CMOS IMAGE SENSOR as surveillance system in various fields.

In this paper, a literature survey was conducted on CMOS image sensors’ predominant applications helping humanity in different fields. These fields include automotive, internet of things (IoT), intelligent surveillance systems (ISS), space, the military, and wireless sensor networks (WSNs). The comfortability with and reliance of human beings on these applications are rapidly increasing day by day, as shown in Figure 2 and Figure 6.

### 3.1. ISS (Intelligent Surveillance Systems) CIS Applications

Surveillance systems are being part of human lives for safety and security purposes to avoid thefts, attacks and help police departments catch culprits or burglars. However, the cameras cannot be placed in restrooms due to privacy issues, and due to this reason, old age people falling accidents cannot be monitored. Most people feel discomfort about video recording cameras in open public places, so privacy preservation policies are recently requested by people. It is hard to identify the differences between regular cameras and privacy preservation cameras. Nakashima et al. [10] developed a privacy preservation sensor for person detection to identify the person’s state and position without capturing and images.

A privacy preservation sensor can detect the person’s position by differentiating the background brightness and object brightness in a one-dimensional manner. This sensor can detect the person fallen or standing by keeping it in a vertical position and can identify the person’s position by keeping it in a horizontal direction.

**Figure 7:**
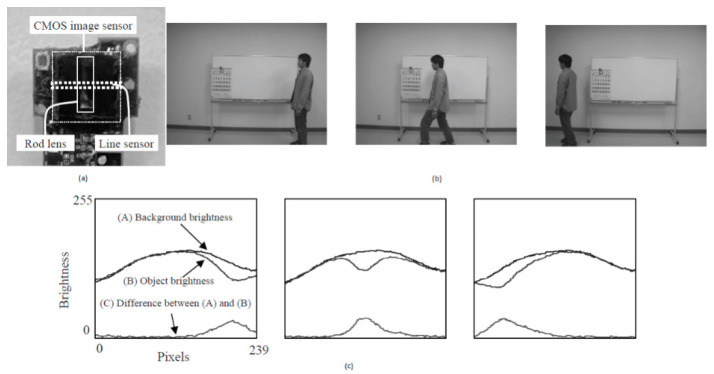
(**a**) Privacy-preserving sensor for person detection; (**b**) Field experiment: person detected at the right middle and left side positions; (**c**) The brightness distributions are made according to the position of the person. Adapted with permission from [10] Elsevier, 2010.

Using two sensors in two directions, one can detect both position and the state of the person, as shown in Figure 7.

**Figure 8:**
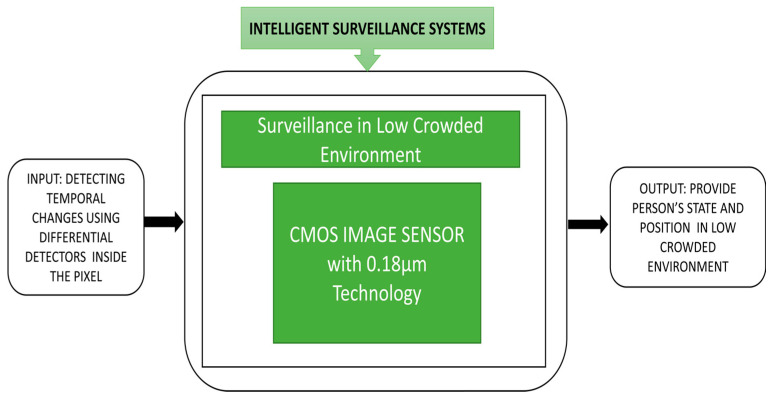
Surveillance in low crowed environment. Empty boxes and illegible text.

Habibi et al. [11] developed a low-power smart CIS suitable for low crowded environments. The sensor captures the images and detects the image’s temporal changes using differential detectors inside the pixel. Person detection in the system can be done by considering pixels with zero difference and will have a high pixel value with the person’s edges entered into the frame. The change window inside the circuit will identify the large temporal changes in the pixels and observe its black box approach in Figure 8.

Pham et al. [12] presented a visual surveillance and intrusion detection application network in which small CIS cameras are connected to sensor nodes to obtain visual data for rescue operations, intrusion detection and search operations. Different visual image sensor boards were introduced such as Cyclops [13], Citric [14] SeedEyes [15], Panoptes [16], iMote2 [17], FireFly [18], Eye-RIS [19], WiCa [20] which runs on ad-hoc networks.

**Figure 9:**
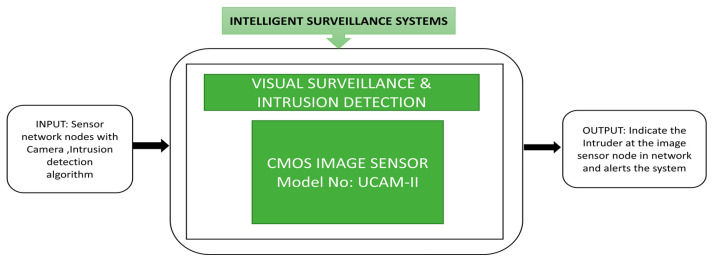
Visual surveillance and intrusion detection application network. Empty boxes and illegible text.

This image sensor node uses an UCAM camera for image capture, and a wireless sensor network can indicate an intruder at the image sensor node, using the black box approach shown in Figure 9.

**Figure 10:**
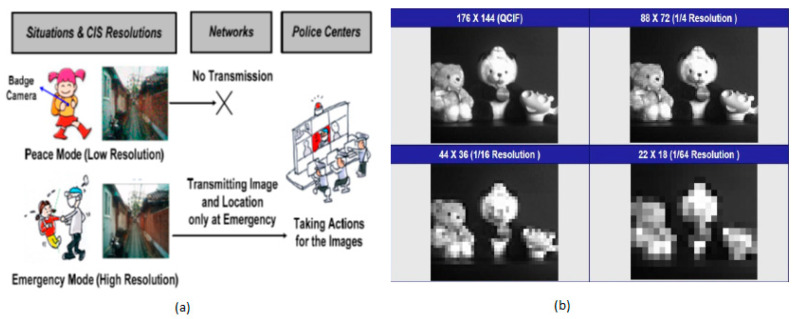
(**a**) Surveillance modes-peace mode, emergency mode; (**b**) Images of different resolution modes [21].

Kim et al. [21] developed the multi-resolution mode CIS for ISS applications shown in Figure 10. This imager can configure its image resolution to a lower level in less monitoring mode and adjust to higher image resolution in high monitoring or secure mode. Thus, adaptability in resolution can save a lot of power for the surveillance system, which is always ON.

**Figure 11:**
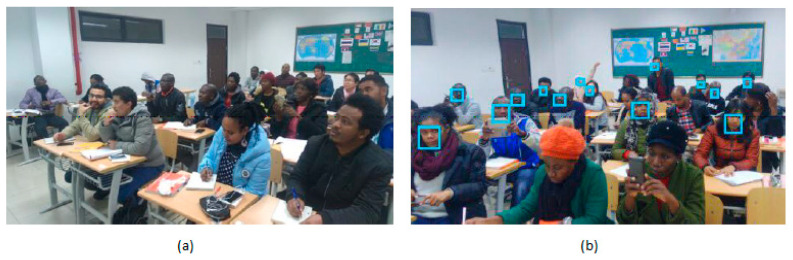
(**a**) Image captured in classroom; (**b**) Captured facial expressions detected using FER technology [23].

Kumagai et al. [22] proposed a back-illuminated stacked CIS which detects moving objects under different lighting conditions. This imaging sensor has a real-time moving object detection function limited to a predefined range. The event-driven sensor in the imager provides event recording and consumes less power and less bandwidth when operating in low power sensing mode. Emotions and motivations are the two key factors that resemble a positive learning environment. The positive emotion always comes from supportive teaching and good interaction between teacher and students. Learning engagement is a positive classroom emotion, but it is tough for the teachers to monitor the students’ emotions. Boonroungrut et al. [23] investigated classroom emotion by using a facial emotion recognition (FER). FER is a technology with cloud-based FER application program interfaces for real-time social interactions. Different types of facial expressions like happiness, sadness, surprise, fear, neutral, anger, etc., are detected, and this monitoring was conducted for five weeks, as shown in Figure 11.

Nowadays, traffic jams are becoming a severe problem in metropolitan cities, so various monitoring systems have been introduced to analyze urban development traffic behavior. Freeman et al. [24] developed a novel technique to monitor and capture stable vehicles’ images at two different junctions at different times in a day.

**Figure 12:**
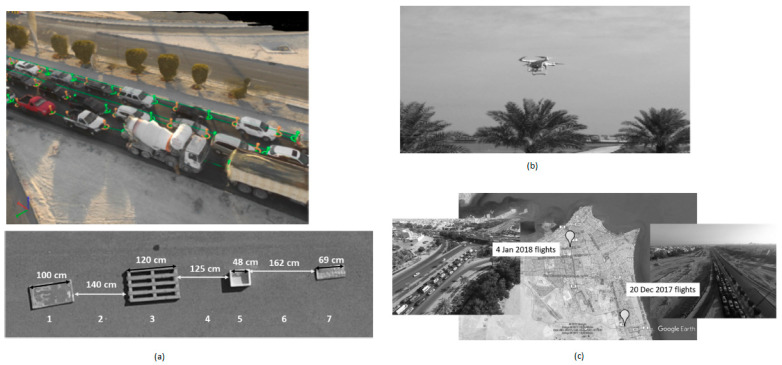
(**a**) Captured image of traffic on the Kuwait highway; (**b**) Drone Flight; (**c**) Flight locations in north and south Kuwait [24].

Using this system, the traffic will be easily monitored, and vehicle emissions that affect the air quality can be detected without using observers to count the vehicles and classify them in different places. This drone flight is a part of an intelligent traffic system. These flights are sent to the north and south highways of Kuwait and the distance between the vehicles observed and calculated. They are thereby categorized with respect to their year, model, and manufacturer to predict the presence of vehicles for air quality monitoring purposes as shown in Figure 12.

Nuclear energy is a rapidly growing technology in the current world. Numerous experiments have been conducted to detect nuclear radiation evolving from nuclear experiments. Nuclear radiation can be detected by using gas detectors, semiconductors, and scintillation detectors. The importance of nuclear radiation detection is obvious, and it has been used in airports, seaports, and land border check posts. Usually, dedicated radiation detection systems are expensive and cannot be kept in public places. CIS plays a prominent role in nuclear radiation detection.

**Figure 13:**
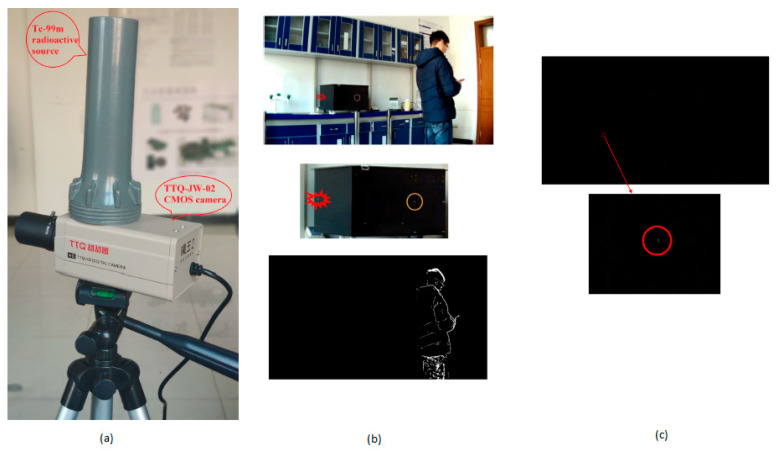
(**a**) CMOS camera used for nuclear radioactive signal detection; (**b**) Field experiment; (**c**) Radiation bright blotch. Adapted from [25] with permission of Elsevier, 2020.

Yan et al. [25] developed an uncovered CMOS camera that can detect nuclear radiation while working in surveillance mode, as shown in Figure 13a. Videos are recorded using this camera with a volunteer person wandering around the room under the camera’s view. Bright blotches were detected due to the radiation particles exciting the eletrons and can be seen as blotches in Figure 13b,c. Whenever the electrons are excited due to the radiation particles and CMOS sensor visible light, bright blotches are captured by using the CMOS camera.

By introducing CIS into the medical field surveillance, a drastic development change is made in monitoring neonatal, premature babies who require continuous monitoring until their organs in the body achieve normal operation. Many techniques were already implemented to monitor neonates by sticking the invasive electrodes on their body, which can cause skin infections later.

**Figure 14:**
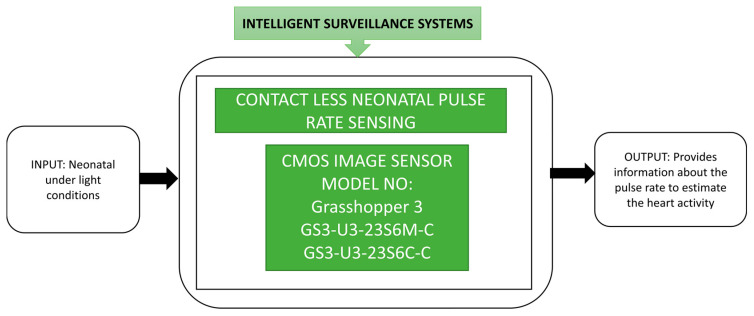
Non-contact neonatal monitoring system. Empty boxes and illegible text.

To avoid this, Paul et al. [26] suggested a noncontact sensing method for pulse rate detection, represented in the black box approach shown in Figure 14, in which neonatal monitoring is performed by using CIS-based video cameras and light. This contactless monitoring to analyze the vital signs of neonatal can be done remotely. In this phenomenal monitoring unit, premature babies will have more advantages by avoiding the cables and invasive electrodes on the body.

**Figure 15:**
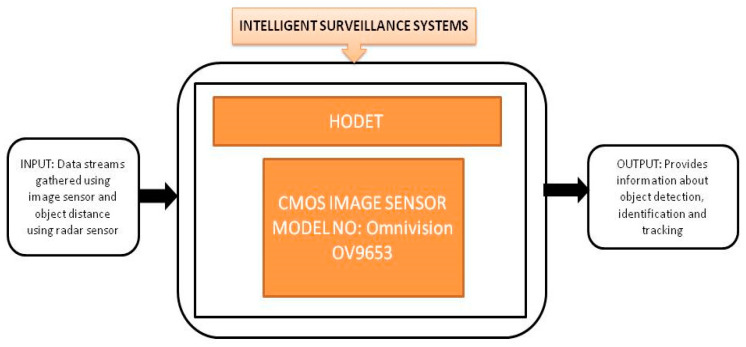
Hybrid Object DEtection and Tracking (HODET).

Demand has increased for proficient, effective, and dependable monitoring systems for surveillance to sustain the situational awareness in military missions, public safety, battlefield monitoring, and natural disaster early detection monitoring and recovery. Optical video surveillance is not a suitable approach to monitor such applications though it is popular. st. Cyr et al. [1] proposed a new Hybrid Object DEtection and Tracking method named “HODET” using millimeter-wave radar, a visual sensor. It is a computing device to perform object detection, identification, and tracking by using image sensors and radar sensors simultaneously. The radar sensor will give an exact distance, whereas the camera can estimate the object distance illustrated in the black box approach shown in Figure 15.

### 3.2. Internet of Things (IoT) CIS Applications

Over the past decade, a familiar term in all developed countries, and that people rely on it in most essential fields is the “internet of things” (IoT). IoT is a developing informational network that can connect a group of sensors to perform multiple tasks. It is not science fiction or an unnatural thing happening in the industry. It works by using the technical improvements and advancements of various standard devices combined to form a network. For example, shortly, weather jackets will be introduced with an amazing feature that can make the jacket warm in cold weather and cool the hot weather jacket. This is achieved automatically by gathering weather information and sensing the body temperature to maintain the required body temperature by providing hot or cold conditions. A few other existing examples are driverless vehicles, and voice-assisted controlled home appliances.

**Figure 16:**
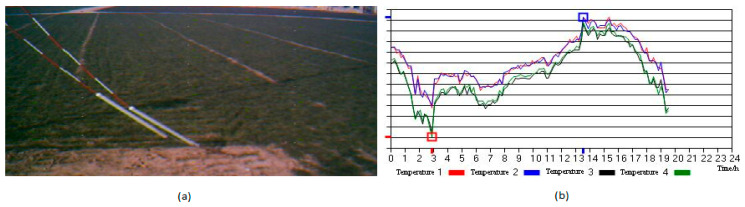
(**a**) Captured image of crop monitoring network; (**b**) Temperature curve changing with humidity. Adapted from [28] with permission of Elsevier, 2011.

There is a strong reason to use the IoT in the agricultural field, i.e., lugens is one of the rice pests that damage rice crops on a very large scale in the rice-growing countries of Asia. The rice crop is a crop that is continuously grown in all seasons. Due to lugens every year around one million tons of rice are spoiled. IoT technology is applied to monitor *Nilparvatha lugens* which transmits viruses like grassy rice stunt and ragged rice stunt. To monitor *Nilparvatha lugens*, Cai et al. [27] developed a wireless sensor node in the IoT to perform automatic data collection, make real-time decisions, and transmit functions. An OV6620 CIS module is used as a camera module to capture the wireless sensor node design images. As traditional agriculture is changing into modern agriculture today, the IoT plays a crucial role in information gathering and creating wireless sensor networks in the agricultural sector. Zhao et al. [28] proposed a wireless sensor network for agriculture applications. Two types of nodes are implemented and system networking is accomplished in a crop monitoring system. This network was deployed in Beijing and gathered the temperature changes with respect to humidity for 24 h after switching on the monitoring system as shown in Figure 16.

**Figure 17:**
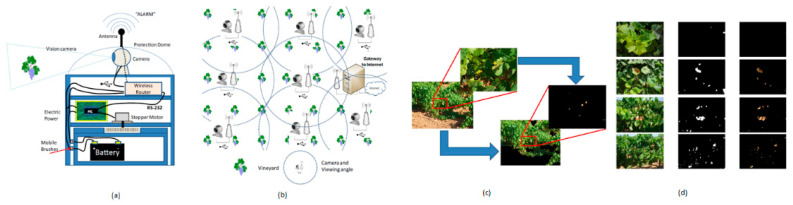
(**a**) Sensor node for a vineyard monitoring system; (**b**) Vineyard monitoring by cameras in a wireless sensor network; (**c**) Detection of brown leaves in vines; (**d**) Captured images of brown leaves with different sizes taken from different distances [29].

Lloret et al. [29] developed a wireless sensor network to monitor vineyard fields. Each sensor node of the wireless sensor network will take images of the field and process the images internally using image processing techniques to identify leaves’ uncommon status. Usually, pests, diseases nutrient deficiencies can cause the leaves to appear unusual. If the leaves are identified as defective, then the sensor node will send a message to the sink node via the wireless sensor network to alert the farmer about the plants’ problematic status. The diamine putrescine or leaf roll can cause considerable damage to grapes, as shown in Figure 17.

**Figure 18:**
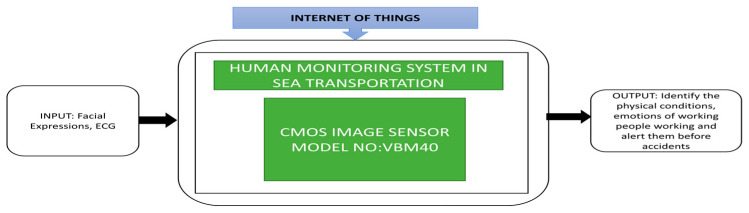
Human monitoring system in the real ship on board monitoring different emotions of the navigational officer.

Every year accidents happen in sea transportation due to several reasons like crew drowsiness, mishandling of steering and lack of attention due to crews sleeping at sea traffic. Arima et al. [3] developed a human monitoring system for sea transportation safety. This system aims to identify any small changes in the physical conditions of the people working on the ship during the trip and alert people before the accidents. Facial expressions and electrocardiogram (ECG) data are the key components of this human monitoring system. This system was named the Kinetic Information Integrator (KII) system. This system involves various network cameras and a KINECT sensor, capable of identifying the posture and position of the person’s skeleton in three dimensions. Its black box approach is shown in Figure 18.

The face of the navigation officer is monitored during field operation by the KII system. The officer is walking around in the navigation ship to ensure the ship’s safety. Network cameras can sense the officer’s facial expressions while he is in the sensing range.

**Figure 19:**
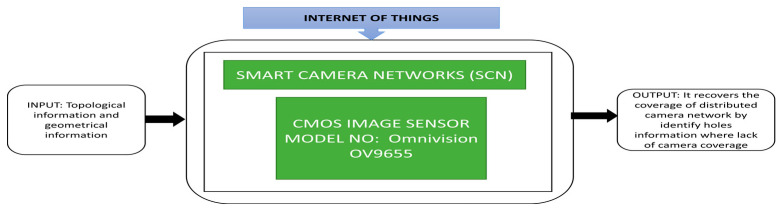
Smart camera networks-based surveillance system with CITRIC mote.

Smart camera networks are a newly evolved category in sensor networks that supports high power in network-enabled signal processing. Chen et al. [30] described a low bandwidth wireless camera network platform named CITRIC, suitable for distributed video and image processing and explained its usage in smart camera networks. In the experimental trials, a testbed is used and smart cameras were connected in every contour. The CN complex concept helps identify the path by communicating with the cameras attached at the wireless camera network’s contours. A remote control car was sent into the layout and a path obtained by deploying the network to test it. Its black box approach is shown in Figure 19.

**Figure 20:**
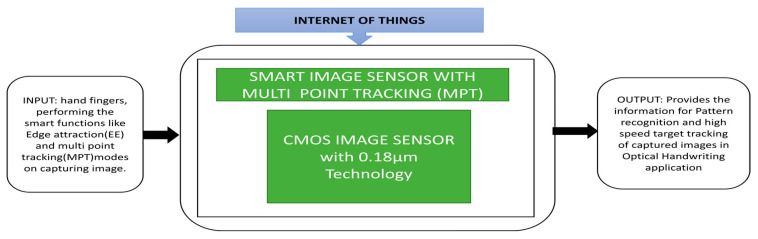
Smart image sensor with Multi Point Tracking (MPT).

Yin et al. [31] presented a smart image sensor with Array Level Image Signal Processing (ALISP) and Event-Driven Peripherals (EDP) to achieve multi-point tracking (MPT) with edge extraction. The authors presented a prototype setup for an optical handwriting recognition application. The demonstration is done using the letter I in the Chinese language by using the hand fingers, and the image obtained captured by the smart sensor is displayed on a computer by applying the edge extraction technique with the black box approach shown in Figure 20.

**Figure 21:**
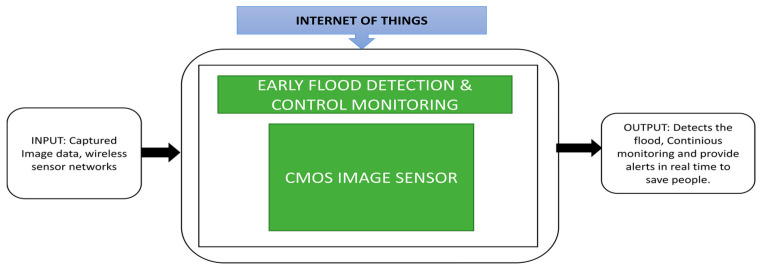
Flood detection and control monitoring system.

Natural disasters like floods occur every year, and due to lack of immediate rescue actions, the rate of peoples’ deaths is increasing year by year. During floods, water dams can overflow suddenly into nearby living areas. People often cannot get evacuated immediately, which leads to the sudden death of hundreds of people. Thekkil et al. [32] presented a wireless sensor network-based early flood detection control monitoring system. This system uses CMOS image sensors to gather data in the form of images and transmits these images via wireless sensor nodes to remote monitoring stations using Zigbee and Global System for Mobile (GSM) communication networks. Clients will get an alert from the remote station to take the necessary action to save people in the flooded area. The black box approach is shown in Figure 21.

The proposed system architecture explains how the interaction happens between remote host and client via wireless sensor nodes, Zigbee and GSM networks. A flood detection and control monitoring system is implemented in the Java software Net Beans, which uses a SIFT algorithm to find the dam’s current water level.

Patokar et al. [33] proposed a precision agriculture system design focusing on a monitoring systems that can collect important data, i.e., temperature, soil moisture, humidity, and sprinkler water flow. This data will be sent to a personal computer and necessary decision-making and actions will be done with the help of the internet of things involved. Using information technology in agriculture can help farmers achieve good productivity and soil fertility. Raj et al. [34] developed an automation system using the internet of things that can operate by using voice commands in various languages to control different home appliances. It is a network-based wireless home automation system that uses Google Assistant to communicate users with the devices.

**Figure 22:**
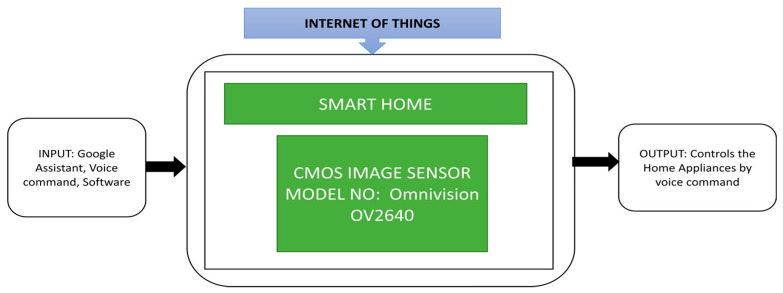
Home automation system.

This system is beneficial for old age people and physically disabled people who cannot easily move. This system can be controlled in two languages, and CIS OV2640 is used as a mini camera on an Arduino board, and its black box approach is shown in Figure 22.

**Figure 23:**
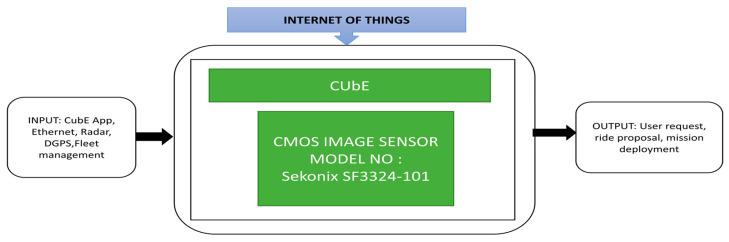
Continental Urban Mobility Experience (CUbE).

As the world population is increasing rapidly, this brings challenges in transport for working offices, inside the large organization’s route map. Hartmannsgruber et al. [35] introduced Continental Urban Mobility Experience (CUbE), a driverless shuttle for cities. It works on the concept of fleet management to communicate the CUbE with the users. This CUbE can transport the people from source A to destination B in the allocated area in their work facility. This fleet concept can help users choose the appropriate CUbE in their pick-up location and drop them off at a target destination by sharing the ride with co-passengers traveling along the same routes. To use this facility, they introduced an android application called “Call A CUbE.” This application can be downloaded from the Android Play store and allows the users to book rides inside the transit area. This application will also indicate the status of available CUbEs in your area concerning trips and timings. Figure 23 presents the black box approach of the CUbE platform, its backend software using fleet management, and an Android application named “Call A CUbE.”

### 3.3. Space CIS Applications

CIS are becoming the crucial component in space applications to measure the distance, altitude, and observing the Earth for various parameters like water, ozone levels, etc. Apart from this, CIS are involved in star trackers, rover cameras, satellite monitoring, space station monitoring, etc. CCDs were widely used in space applications earlier. Due to the high power consumption of CCDs, CIS is however, preferred. CIS has a high dynamic range and modulation transfer function, which are the critical components for space applications.

**Figure 24:**
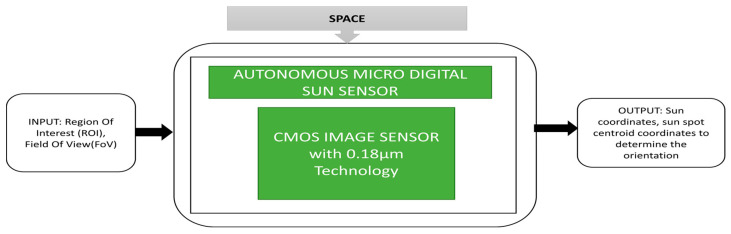
Autonomous Micro Digital Sun Sensor (µDSS).

The attitude information is an important parameter to be monitored for a spacecraft. Sun sensors are a kind of attitude sensor that can detect the spacecraft’s orientation by observing the Sun’s angular position. Xie et al. [36] proposed a Micro Digital Sun Sensor (µDSS) that can be used in microsatellites due to its special functions like low power consumption, radiation hardness, miniature size and high accuracy. The black box approach of a micro digital Sun sensor is shown in Figure 24. This sensor works with two modes of operation, namely acquisition mode and Sun-tracking mode. During the acquisition mode, the Sun’s coordinates can be estimated within the determined region of interest. During Sun-tracking mode, the sunspot final centroid coordinates will be determined. This application is applicable to satellites in low orbit that circle around the Earth in ninety minutes. A cross-view of APS+, which is used as an imager in the micro digital Sun sensor is produced and for radiation tolerance, this sensor is covered with an aluminum shield. Another critical parameter in space applications is lightning detection and imaging in the Earth’s orbit during thunderstorm observation. This observation gives essential data to estimate the changes in the climate. To analyze the origin of nitrogen oxides origin, lightning should be monitored. The detectors on the ground have an insufficient range which causes less coverage of low-density population regions and oceans.

**Figure 25:**
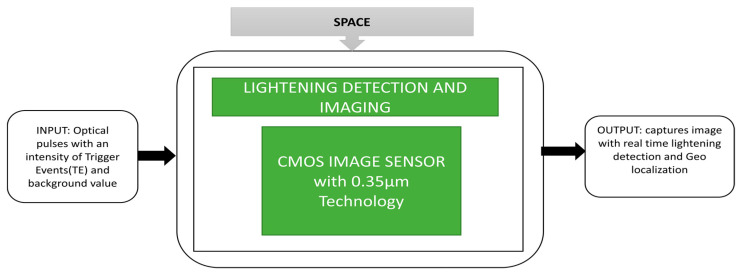
Lightning detection and imaging observation over earth.

Rolando et al. [37] presented a dedicated CIS for lightning detection and imaging, and evaluated the image sensor chip potential used for lightning detection. The lightning imaging sensor instrument and the imager satellite are dedicated to monitoring lightning in the Earth’s atmosphere. The lightning imager continuously observes and achieves above 80% coverage of the Earth’s globe and provides real-time lightning detection and geolocalization, and its captured images. The corresponding black box approach is clearly illustrated in Figure 25.

Star tracking is one of the space applications to track the stars and their radiation measured by their visible light magnitude. Many changes are being made in space technology in terms of micro- and macrosatellite operations. The satellites dedicated to Earth observation require an automatic attitude control system to drive the satellite to the required destination. Many navigation sensors like gyrosensors, Sun sensors, observation sensors having imagers, star trackers, and Earth magnetic sensors are involved among which star trackers are the most suitable ones. Qian et al. [38] developed an architecture for CIS to apply an adaptive integration time function where brighter pixels are detected and read quickly. Low-intensity pixels will take a long integration time to be detected.

**Figure 26:**
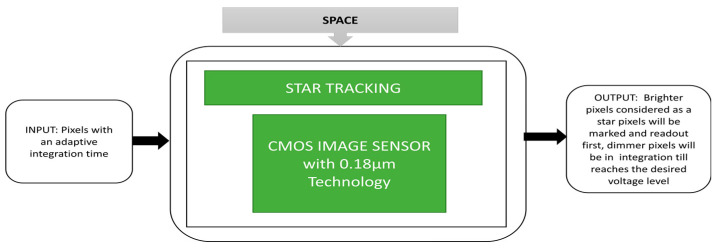
Imaging camera setup for star tracking measurement.

The CIS used here is to capture the starfield scenes where stars are considered the brighter pixels and detected instantly. Its black box approach is described in Figure 26. This imager can prevent saturation and capture images with low power consumption due to its adaptive integration time feature.

**Figure 27:**
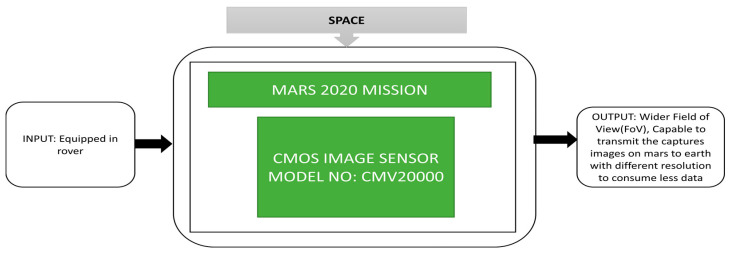
Enhanced Engineering camera (EECAM) using CMOS image sensor CMV-20000, i.e., Navcam.

Mckinney et al. [39] proposed an enhanced engineering camera (EECAM) to be used in the 2020 Mars Rover mission. This rover is incorporated with the next-generation system, an upgrade to the earlier engineering cameras used in the Mars Science Laboratory Rover mission and Mars Exploration Rover mission. Earlier generation Hazcams and Navcams were used as engineering cameras in the Mars Exploration Rover mission during 2000. The Mars 2020 enhanced engineering cameras use a 20 MP color CIS instead of the CCDs used in earlier Mars missions, and its black box approach is illustrated in Figure 27.

There are two cameras incorporated into Mars 2020 rover, namely a Navcam and a Hazcam. The Navcam is mounted on a pan/tilt mast and used to capture color stereo panoramic images from two meters height from the Mars surface, whereas the Hazcam is mounted on the rover body and can capture regular color stereo images from a height of 0.7 m above the Martian surface. A new camera called Cachecam is introduced and can capture sample material images. The greater number of EECAMs being incorporated into the Mars missions will increase the total operating efficiency of the 2020 Mars rover on the Martian surface. This launching of the Mars mission was scheduled for the summer of 2020.

**Figure 28:**
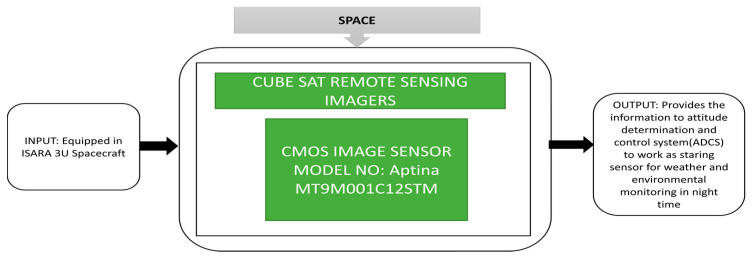
NASA Integrated Solar Array and Reflectarray Antenna (ISARA) mission.

The building of satellites is becoming a critical task for scientists to monitor fundamental components in a rocket and examine the functionalities of each block of the satellite. It increases the manufacturing cost and operating risks during maintenance, but the functionalities are split to reduce costs and balance the risk in operation for CubeSats. Pack et al. [40] described two CubeSat remote sensing missions, which were proposed by the Aerospace Corporation. The Aerospace Corporation developed the CubeSat Multispectral Observation System (“CUMULOS”) and R3 sensors, which can be easily incorporated on 3U Cubesats. CUMULUS is used to do environmental and weather missions. Over 19 years, the Aerospace Corporation has launched 20 picosatellites and nanosatellites and has yet to launch a few more in completion stages. CUMULOS is a three camera payload incorporated into the NASA Integrated Solar Array and Reflectarray Antenna (ISARA) mission. It is a mini weather satellite that focuses on low light conditions and the R3 sensor is a CubeSat which focuses on operational land imager instruments. The complete structure of the ISARA spacecraft with antennas and payloads containing three cameras, and its black box approach are clearly explained in Figure 28. There is no additional space required to insert the CUMULOS payloads in the spacecraft, the R3 satellites line of assembly and regions are covered by three CUMULOS cameras simultaneously.

The primary purpose of CUMULOS is to be a staring sensor and having three cameras, which can capture one frame from each camera simultaneously with the other two cameras to scan the required region. These two sensors, CUMULOS and R3, can integrate the more complex payloads on CubeSats to conduct remote sensing research.

**Figure 29:**
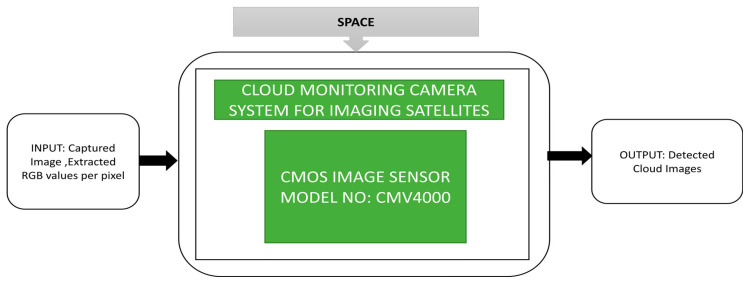
Cloud monitoring camera system for imaging satellites- INSAT satellite and NOAA GOES satellite.

In the part of remote sensing research activities, Vala et al. [41] proposed a camera system for cloud monitoring designed to be incorporated in remote sensing satellites. Using this system, clouds’ presence can easily be detected, which is useful for applications like weather, oceanography, disaster assessment and geology, etc. The satellites’ captured images get contaminated and transmit false information to the Earth stations due to clouds. The proposed cloud monitoring system is miniature and consumes less power. This system behaves like a secondary camera system that can identify clouds’ presence and a corresponding decision will be sent to the primary camera to turn ON or OFF. The hardware unit of the detector head assembly and the camera electronics are incorporated to form a cloud monitoring system. The proposed cloud detection algorithm is implemented on INSAT satellites, NOAA GOES satellites. The system’s future development task is to identify the thickness of the clouds and its black box approach is shown in Figure 29.

CMOS image sensors have become one of the essential blocks in space applications like remote sensing satellites, Earth observation satellites, rovers, etc. Earlier CCDs were used as imagers for these applications due to their low noise and high image quality functions but CCDs consume more power, are big in size, and not tolerant of what? Due to these drawbacks of CCDs, CIS are chosen instead. Kim et al. [42] proposed a pixel design that can enhance CISs’capacity without including additional photocharge in the predefined pixel area. A known fact is that the damage caused by the radiation effect on CIS will be decreased by shrinking the CMOS technology to deep submicron size and reducing the thickness of the oxide layer.

**Figure 30:**
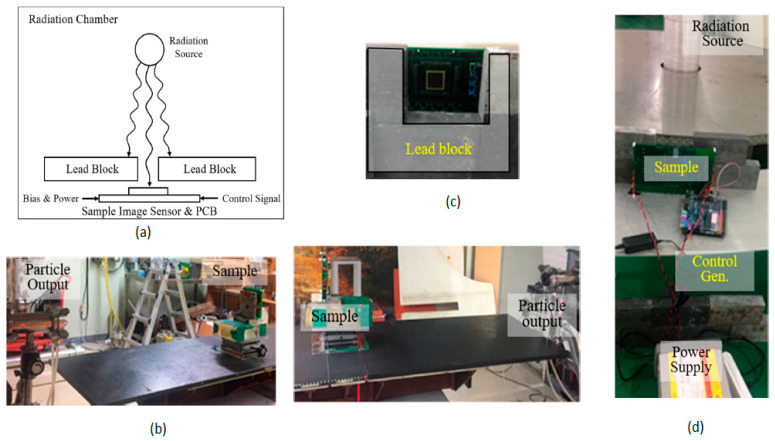
(**a**) Radiation test setup block diagram; (**b**) Displacement damage dose test with metal shielding(first image) and without metal shield (second image); (**c**) Total ionizing dose test setup front view; (**d**) Total ionizing dose setup with radiation source [42].

Two tests are performed to find the radiation tolerance for the proposed imager, the displacement damage dose (DDD) test and total ionizing dose (TID) test. DDD test is conducted with metal shielding to protect the remaining electrical components (first image) and without metal shield (second image) as shown in Figure 30b. A total ionizing dose (TID) test is conducted by usinggamma radiation as a radiation source, as shown in Figure 30c,d. By performing these two tests, this imager is radiation tolerant and suitable for use in space applications.

It is essential to know the information of asteroids, including their shape, surface composition, internal structure and surface morphology, to know their origin and evolution. Around 14 types of asteroids have been found and assessed so far by nanospacecraft such as CubeSats. Nanospacecraft can characterize and provide detailed information about various asteroids in a mini time frame by sending many spacecraft at a time to monitor numerous targets.

**Figure 31:**
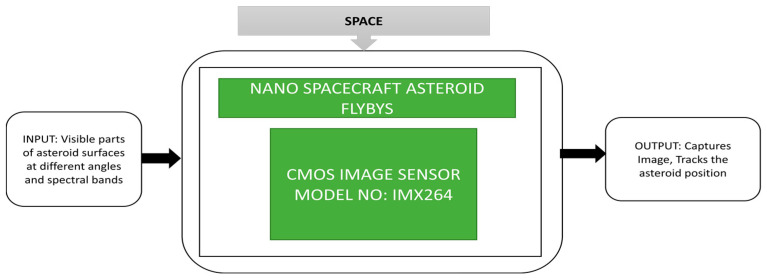
Spacecraft for Multi Asteroid Touring (MAT) mission.

Pajusalu et al. [43] developed a prototype and simulated a nanospacecraft virtually to characterize the asteroids. The multi-asteroid touring (MAT) nanospacecraft mission is an example of this approach. The corresponding design used in MAT nanospacecraft missions and the prototype captured images of the moon, and its black box approach is shown in Figure 31.

Due to daily human activities, there is a gradual increase in the concentration levels of greenhouse gases in the atmosphere, leading to drastic changes in the climate. This climate change is influencing the water content levels on Earth in most of the areas very severely. Due to this effect, the productivity of crops is also affected to a great extent. CubeSats [44] could be used to monitor the levels of greenhouse gases. Jallad et al. [4] described the MeznSat, a 3U CubeSat which carries a shortwave infrared (SWIR) microspectrometer as its primary payload to detect the greenhouse gas levels in the atmosphere. The two familiar greenhouse gases that exist in the atmosphere are carbon dioxide and methane. Due to its high heat absorption property, methane cannot stay longer in the atmosphere than carbon dioxide.

**Figure 32:**
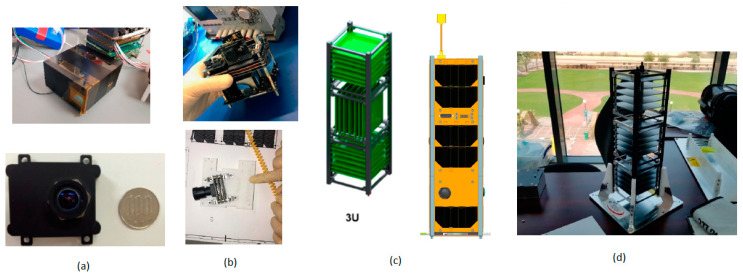
(**a**) Argus2000 Spectrometer with CIS based RGB camera; (**b**) Attitude determination and control subsystem (Upper Image) and star tracker (lower Image); (**c**) 3U CubeSat platform and its solar cell distribution; (**d**) Mechanical structure of MeznSat [44].

To find the nutrient concentrations in the sea of the Arabian Gulf, algal boom occurrences could be predicted by using a CMOS image sensor-based RGB camera in the shortwave infrared region. The primary objective of this MeznSat is to identify the carbon dioxide and methane levels in the atmosphere with the help of a shortwave infrared region spectrometer. This satellite’s primary payload is the Argus2000 shortwave infrared spectrometer, and the secondary payload is a CIS-based RGB camera shown in Figure 32a. The mechanical structure of Mezn Sat, which is a 3U CubeSat with its solar cell distribution representation, is shown in Figure 32c,d. This satellite was initially scheduled to launch in March 2020 and launched successfully on 28 September 2020 from Russia, and it is a UAE’s most prestigious national project. Small satellites and CubeSats can increase the availability of astrophysics measurements, but an astrophysical demonstration is needed to realize their potential before any measurements.

**Figure 33:**
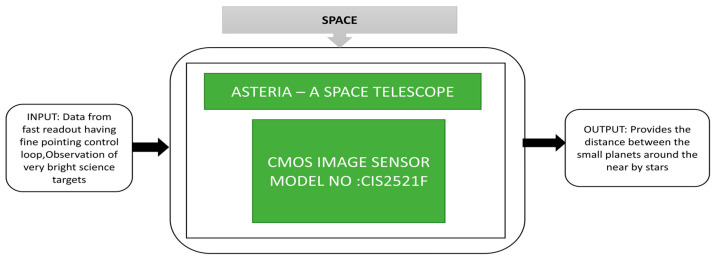
Arcsecond Space Telescope Enabling Research in Astrophysics (ASTERIA).

Knapp et al. [45] explained the 6U CubeSat space telescope-Arcsecond Space Telescope Enabling Research In Astrophysics shortly called ASTERIA demonstration for high precision photometry. The mission of ASTERIA is to launch a demo to measure the distance between small planets that are present around nearby stars. Various missions were conducted in space to determine the space parameters like types of stars, planet evolution, and magnitude of stars. The Transiting Exoplanet Survey Satellite (TESS) mission [46] was launched in 2018, did a survey about the total sky for a series of observations for one month. The Planetary Transits and Oscillations of stars (PLATO) mission [47] with 26 small telescopes are used to analyze the sky’s bright stars. ASTERIA is a small space telescope with higher photometric precision than available ground telescopes and a device to perform bright star observations. The payload of ASTERIA is a combination of an optical telescope and electronics. The CMOS image sensor acts as an imager in the optical telescope. The layout and assembled ASTERIA satellite and its black box approach is shown in Figure 33.

A CMOS image sensor is used as a science detector in ASTERIA due to its fast readout mechanism compared to CCDs. Another additional feature of ASTERIA is the fault protection system, which protects the battery from undervoltage situations. It can also reach the target star, start observation, and come back to an attitude of Sun point within 43 min after observation with the help of a backup battery.

### 3.4. Military CIS Applications

**Figure 34:**
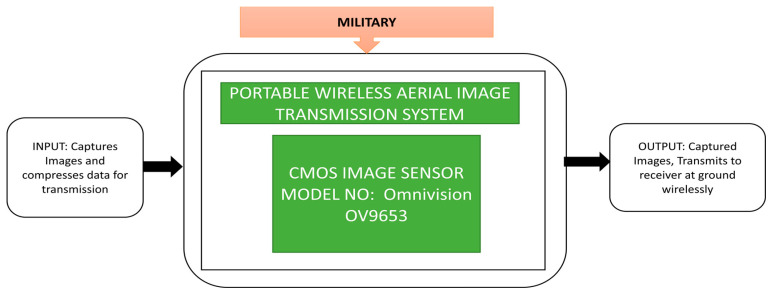
Portable wireless aerial image transmission system.

CIS is becoming crucial in military applications like aerial surveillance, battlefiled monitoring, target recognition, missile detection, etc. Wireless aerial image transmission is one of the key modules in war zones. Zhang et al. [48] developed a portable wireless aerial image transmission system by using an unmanned air vehicle, the CIS OV9653, to capture images, work using an advanced blackfin BF531/533 digital signal processing system, and its architecture with operation is explained terms of its black box approach in Figure 34.

**Figure 35:**
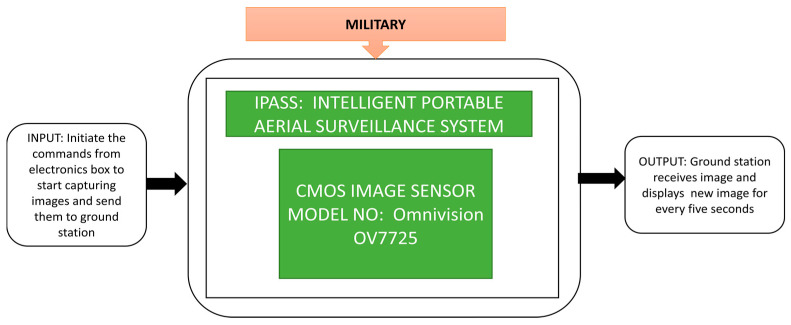
Intelligent Portable Aerial Surveillance System (IPASS).

The BF531 will compress CIS’s images, whereas the BF533 receives and monitors the images on the ground by sampling the data in real-time. It is well suited for field reconnaissance applications and can also monitor bad weather situations. Due to the high demand for aerial surveillance in the military field, especially to know aerial information, including soldier counts, equipment, weapons, etc., unmanned aerial vehicles play a pivotal role in fulfilling military needs aerial surveillance. Blumenau et al. [5] developed an intelligent portable aerial surveillance system termed IPASS, which black box approach concept shown in Figure 35. IPASS is a portable, reliable, user-friendly system that can send aerial images to a ground control station using wireless transmission. IPASS also met the designed specifications, including a maximum reachable height of 100 feet, surviving a fall from 30 feet, and has a wide vision, location detection, image, and local wireless data transmission capability up to 200 feet.

**Figure 36:**
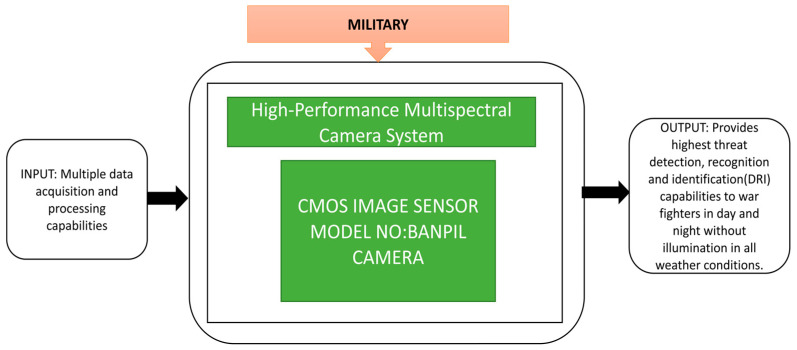
Banpil Multi-Spectral Camera.

This system helps fighters in the battle zone collect visual information and transmit the data wirelessly within 120 feet. Odour et al. [49] explained the low-cost multispectral camera developed by Banpil Photonics (city, state abbrev if USA, country). It is a high-performance short wave infrared imaging camera, most suitable for military applications like threat detection during the day and at night, in all weather conditions. Its black box approach is explained in Figure 36.

**Figure 37:**
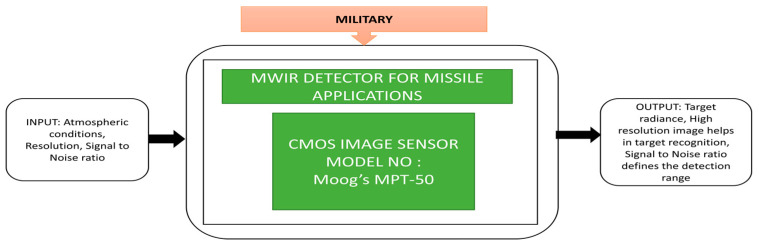
Mid wave infrared imaging detector for missile applications.

This camera had multiple functionalities like data acquisition and processing to support soldiers in threat detection, recognition, and identification (DRI) capabilities to ensure situational awareness. Thermal imaging is also an advanced technology used in defense applications. It can detect an object with thermal contrast from the background without using natural light or other light sources. Kurum et al. [50] proposed a technique similar to binning, a well-known function used for mid-wave infrared imaging detectors to get a good signal-to-noise ratio. An experiment was conducted on a missile system to evaluate the pixel pitch and resolution, which need many of these imagers. Resolution and signal to noise ratio are the two parameters to calculate the detection range and recognition and identification of targets. These two factors are compared in binning enabled and disabled conditions of image acquisition. The most critical missile and weapon detection parameter recognizes the target and avoids damage to the neighboring ships or civilian ships across the sea. A higher resolution is required to identify the target among them. The captured images of a civilian ship and no warships were detected, and the object is identified with the target in red color on a background in blue using the thermal imager. Its black box approach is shown in Figure 37.

**Figure 38:**
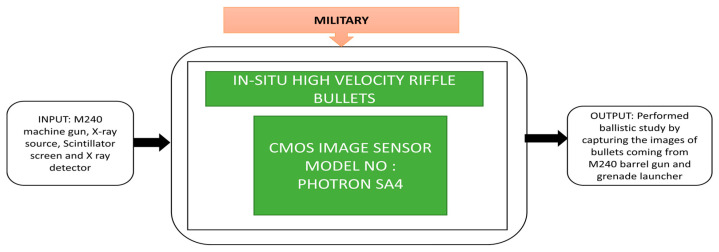
Ballistics experiment using X-ray imaging with a grenade launcher and M240 barrel gun.

The study of ballistics is the background for live-fire testing in the army training areas to maintain safety measures. The study of ballistics is classified into the interior, exterior, and terminal ballistics. Interior ballistics refers to the gun’s barrel’s internal process when the gun is ignited and till the bullets come out of the barrel. Exterior ballistics describes how the bullet left the barrel with an angle, where terminal ballistics explains what happens after the bullet hits the target. D’Aries et al. [51] presented their experimental results produced from conducting X-ray imaging of rifle bullets fired out of a gun barrel using high frame rate cameras. In this experiment, a CIS is coupled to a scintillator to act as an X-ray image detector. This X-ray imaging has three instruments: a scintillator screen, an X-ray source, and an X-ray detector. Continuous frames of three images were captured at 10,000 frames per second when the bullet is fired from the barrel, and a grenade launcher was also captured while it was firing. The black box approach is shown in Figure 38.

Using wireless sensor networks, wireless image sensor networks are made to provide the observation area’s visual data. Pandey et al. [52] proposed a technique to implement a wireless image sensor that can be used for monitoring and surveillance. This wireless image sensor network has a CMOS image sensor integrated with internal processing and transmission modules. Some of the nodes are connected to radiofrequency, and the rest are connected for Bluetooth transmission.

**Figure 39:**
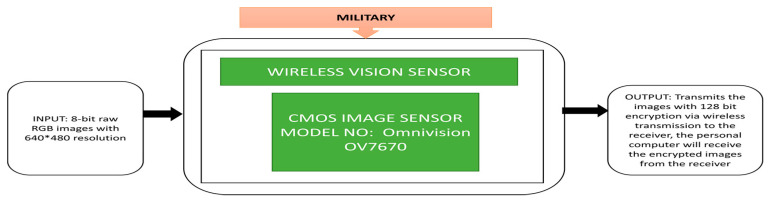
Wireless vision sensor.

The black box approach of a visual sensor node prototype is shown in Figure 39. An image is captured using the CMOS image sensor incorporated in the node internally, and the images are compressed with different ratios to transmit through the XBee communication module.

**Figure 40:**
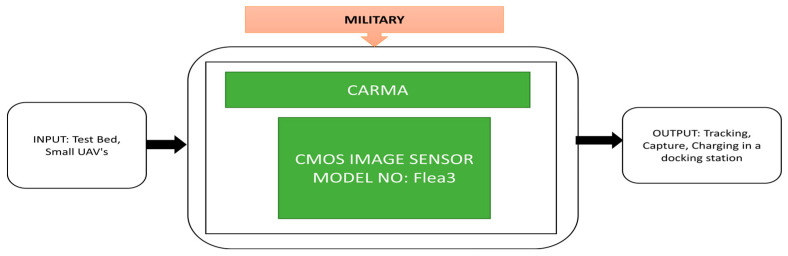
Concept of Catch and Release Manipulation Architecture (CARMA).

Involvement of unmanned aerial vehicles is increasing rapidly in military warfare, surveillance and weather monitoring applications, etc. The US Army has employed a huge collection of categories of unmanned aerial vehicles in the field to perform Intelligence, Surveillance and Reconnaissance (ISR) missions to do search and rescue operations. However, these systems need to land for recharging or refueling. Johnson et al. [53] developed a CARMA similar to the real-time indoor autonomous vehicle test environment (RAVEN), a prototype developed by MIT [54]. CARMA refers to Catch and Release Manipulation Architecture, which enhances the RAVEN applications. This prototype is used to influence manipulation in industries to recharge and capture unmanned aerial vehicles (UAV) which are busy in monitoring and surveillance purposes. The main feature of this system is to capture, track, and charge the unmanned aerial vehicles. The components and architecture of CARMA and its black box approach are shown in Figure 40.

In the process of refueling or recharging the UAVs, the testbed on the ground vehicle will hold the UAVs in the docking station until they get charged. The undercarriage will be a platform for the UAV to safely dock it into the docking station and charging ports will charge the UAV.

**Figure 41:**
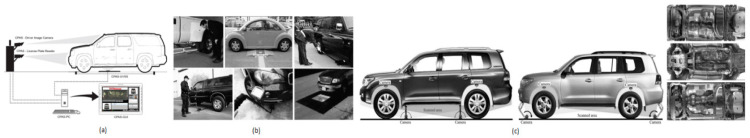
(**a**) Under Vehicle Inspection System (UVIS); (**b**) Real-time Inspection; (**c**) Under view for bomb Inspection [55].

Placing bombs onto vehicles and causing them to explode to create a human loss is one of the major terrorist threats every nation faces now. To overcome this issue, many detection mechanisms are being developed. Majeed et al. [55] proposed an Under-vehicle Inspection System (UVIS) to detect bombs under the vehicles in real-time to monitor and save the people from terrorist attacks. The prominent features of UVIS include good image clarity to show the size, location, shape of the bomb, a clear view of the bomb under the vehicle, a fast processing time to perform the detection task, comfortability, and security. This system can grab the license plate information to detect the suspect or stolen cars and other vehicles using license plate recognition technology. The on-demand screen will help check under the car for bomb inspection and the driver image camera will capture the drivers’ image to match the crime database. The entire Under Vehicle Inspection System (UVIS) is shown in Figure 41.

**Figure 42:**
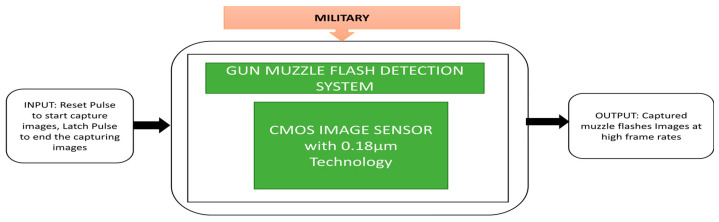
Gun muzzle flash detection system.

Katz et al. [56] developed an architecture involving the CMOS SPAD imager for gun muzzle flash detection. This imager can detect the fast optical and weak signals in very high illumination environmental conditions. Its unique quality is the detection of gunshots quickly in combat fields. The detection system architecture, imager mounted on board, and complete field experiment setup is done in daylight conditions where we will get high illumination. Its black box approach is shown in Figure 42.

During the past decade, the usage of small airplanes is increasing and providing comfort to human living. The misuse of these aircraft causes illegal activities and affects human privacy and country security. Different techniques are introduced to overcome this problem, like net catching, laser weapon shooting and electronic interference, etc. The reconnaissance balloon at high altitude technique is a familiar aerial reconnaissance aircraft with detecting components that are terminated by blasting the balloon tail knot. In this aircraft, laser weapons cannot shoot the target effectively by penetrating the balloon due to surface penetration capability. If the laser continuously targets the balloon’s knot, then it will burn the balloon and thereby destroy the target.

**Figure 43:**
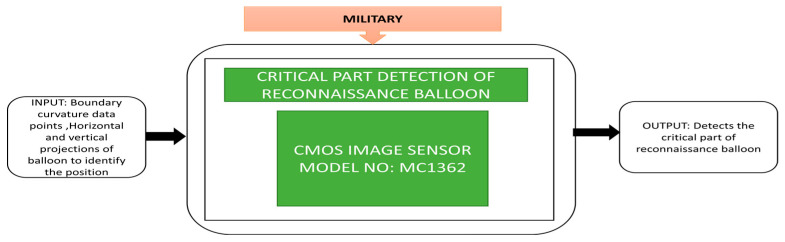
Reconnaissance balloon critical part detection.

Hong et al. [57] proposed an identification method to detect the balloon knot in various illumination conditions and destroy the target. A CMOS image sensor-based detector is used to capture the balloon images under different light conditions with various contrast and blurred values. Field experiments were conducted to identify the knots of white and black balloons under low, high contrast, and medium and heavy blurred lightning conditions. The corresponding black box approach shown in the cited paper is presented in Figure 43.

### 3.5. Automotive CIS Applications

**Figure 44:**
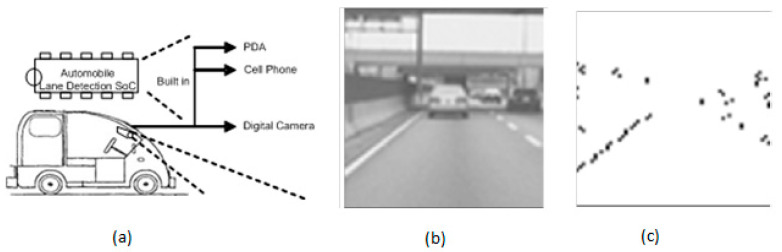
(**a**) Automobile lane detection using CMOS image sensor; (**b**) Original captured image; (**c**) Image captured by CMOS imager [58].

Over the past 20 years, intelligent transport systems (ITS) have grabbed colossal recognition. CMOS image sensors’ role in automotive applications focuses on applications inside the vehicle, vehicle to vehicle, and vehicle and traffic. Inside the vehicle, applications involve capturing real-time images and detecting lanes by the vehicle’s motion. Different lane identification algorithms were proposed from the beginning of the 20th century. Many improvements have been reviewed to meet weather requirements and traffic conditions. The other CMOS imagers developed have mostly focused on high resolution, dynamic range, and noise reduction instead of integrability in compact applications. Hsiao et al. [58] developed a CIS which can capture an image and detect traffic lanes simultaneously. This lane detection system can detect lane markers and capture road images in real-time in different weather conditions, as shown in Figure 44.

**Figure 45:**
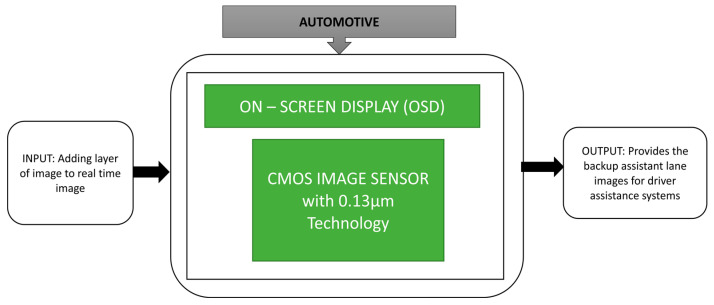
On Screen Display (OSD).

Zhang et al. [59] proposed an on-screen display architecture and SPI interface on CIS used for automobile applications. The backup camera is equipped in the vehicle with the on-screen display embedded in a field-programmable gate array (FPGA) to detect the lanes with the black box approach explained in Figure 45. It achieves a dynamic range of 70 dB.

To meet one of the requirements in advanced driver assistance systems, which monitor the driver’s emotions, Cao et al. [60] developed a time-resolved CIS for non-contact heart detection imager was tested under invisible light and enormous ambient light conditions. An NRI LED array is used to detect the HbO_2,_ i.e., oxyhemoglobin, in human blood. The concentration of oxyhemoglobin is gradually changed with respect to the heartbeat.

**Figure 46:**
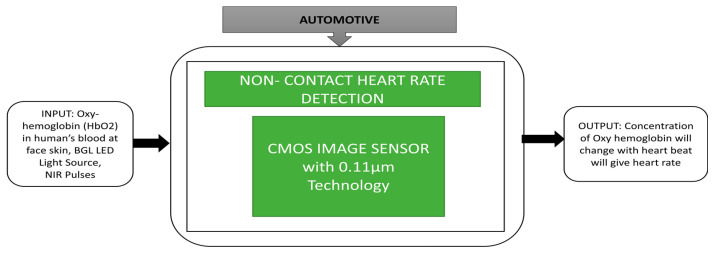
Non-contact heart rate detection of driver during driving the vehicle in motion.

The driver’s facial skin will be an object at a distance of 50 cm from the NIR light source. Cardiac rhythm output was recorded under different bright conditions. The black box approach is represented in Figure 46.

**Figure 47:**
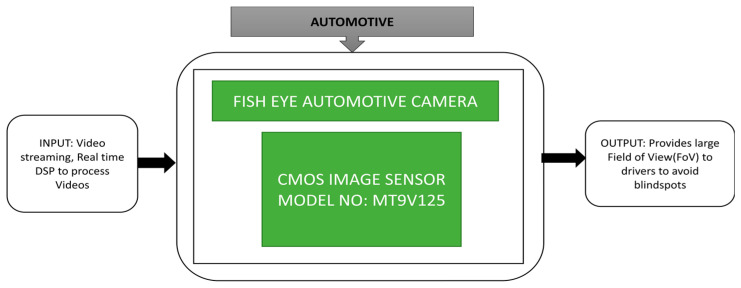
Fish eye automotive camera for blind spot detection.

Vehicle to vehicle communication is a prominent automotive application. Turturici et al. [2] implemented an affordable and flexible embedded system for fish eye automotive cameras in real-time. Fisheye cameras [61] suffer from tangential and radial distortion. It is very crucial to provide accurate vision to the driver by adjusting the captured video of the fisheye camera. Its black-box approach is explained in Figure 47.

Visible light communication utilizes low power light-emitting diodes (LEDs) to provide light and broadcast data in vehicle-to-vehicle communication, a rapidly growing application in intelligent transport systems (ITS).

**Figure 48:**
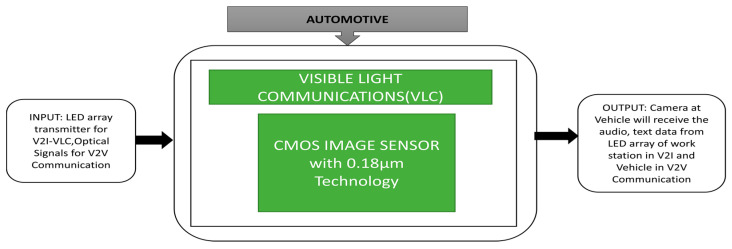
Visible Light Communication (VLC) in two modes of operation namely vehicle to interface (V2I)-VLC using an LED traffic light and a vehicle to vehicle-based VLC System (V2V)-VLC using LED brake lights.

Yamazato et al. [62] introduced two types of communications in automobile applications, namely vehicle-to-interface (V2I) and vehicle-to-vehicle (V2V). A V2I-VLC system field trial was conducted to observe the efficiency under real driving conditions. The black box approach is explained in Figure 48. An LED array is placed on the ground horizontally, and a high-speed camera is mounted on the vehicle’s dashboard. The vehicle is driven at a speed of 30 kmph, and the communication distance ranges between 30 m and 70 m. It has been observed that audio signals are received very clearly up to 45 m distance with no error. In this field trial, there are two vehicles named the lead vehicle and the following vehicle. The lead vehicle has two LED transmitters, a front view camera to take front view images, and a control unit. LED transmitters are attached to the left side, and the right side of the rear window sends an optical signal with 40 degrees inclination. The control unit collects the vehicle’s internal data, such as speed, vehicle id, and data (encoded packets) from LED transmitters. This collected data will be sent to the following vehicle.

**Figure 49:**
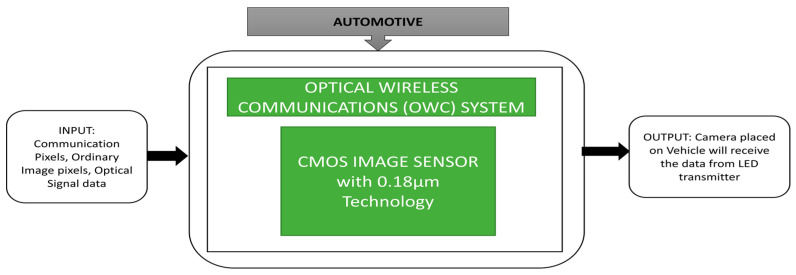
Source identification using image sensor based optical wireless communication system.

Takai et al. [63] developed an image sensor-based optical wireless communication system using a LED as transmitter and a camera as a receiver with a data rate of 10 Mbps per pixel to send colored videos and vehicle internal data, including speed, the status of brakes, etc. It can also communicate with moving vehicles as an additional feature. The black box approach is shown in Figure 49.

Bronzi et al. [64] developed an optical 3D ranging camera using a CMOS Single-Photon Avalanche Diode (SPAD) imager, one of the more prominent drives assistance applications in the automotive sector.

**Figure 50:**
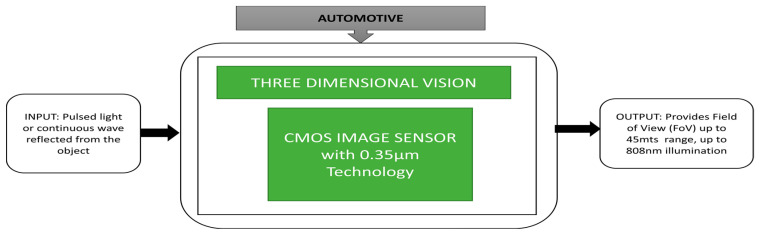
3D ranging CMOS SPAD camera for advanced driver assistance systems.

A 3D ranging camera is mounted on the car to conduct some test trials for two cases: a stable object case and an object in motion case. In the case of a stable object, the images were captured in a parking lot in which the illumination power is enough to make the light hit the target up to a distance of 40 m. We can easily observe the individual’s distance at seven meters and pillars with different ranges and the second case, where the object is in motion when the car is taking a left. The corresponding black box approach is indicated in Figure 50.

Kwon et al. [65] developed a camera-based blind spot detection system for IoT based smart connected cars and replaced the existing radar-based blind-spot detection system.

Three cameras were mounted on the vehicle on the left side, right side, and rear view positions. Continuous monitoring of the three cameras’ images can be seen in the display to avoid the blind spots while the vehicle is in motion.

**Figure 51:**
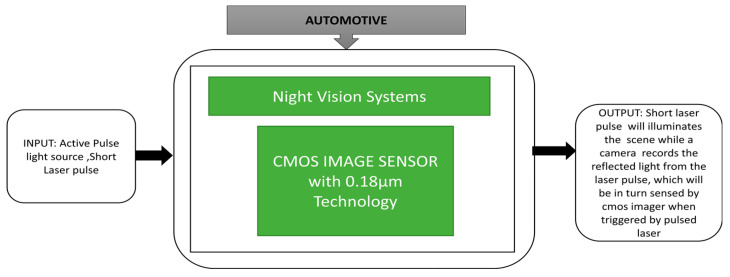
Night vision systems.

**Figure 52:**
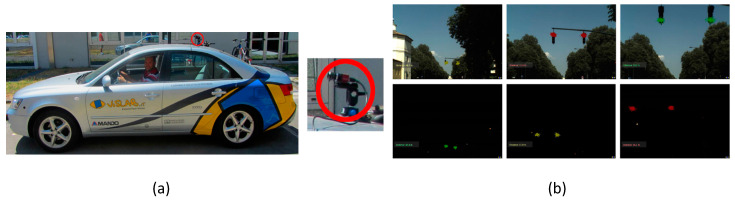
(**a**) Traffic light detection with camera; (**b**) Detection of traffic lights during day and night scenarios. Adapted permission from [67], Elsevier 2015.

Spivak et al. [66] developed a CIS for night vision systems. Its black box approach in shown in Figure 51. It is able to synchronize with an outside light source to extract information. This image sensor provides a wide dynamic range with a minimum of 92 dB. Diaz et al. [67] proposed a unique method to detect traffic lights during day and night environmental conditions and measure distance. This function will be a part of the driver assistance system, which can identify traffic lights with more accuracy from a distance of 10 to 115 m. A demonstration of this method happened on public roads in 2013 in Italy, as shown in Figure 52. Mu et al. [68] proposed an algorithm to identify car path tracking using an open MV in which a CIS is incorporated to recognize the white and black trajectory paths. With this algorithm, a car can select the track of the path instantaneously.

### 3.6. WSN (Wireless Sensor Networks) CIS Applications

Wireless sensor networks play a prominent role in our day-to-day lives by advancing healthcare systems, home automation, temperature control, and environmental monitoring. Among the various WSN applications, those depending on light sensors or temperature sensors produce small data samples. However, changes in WSN technology make these sensors collect more information and integrate CIS to gather huge visible information of the target with less power consumption.

**Figure 53:**
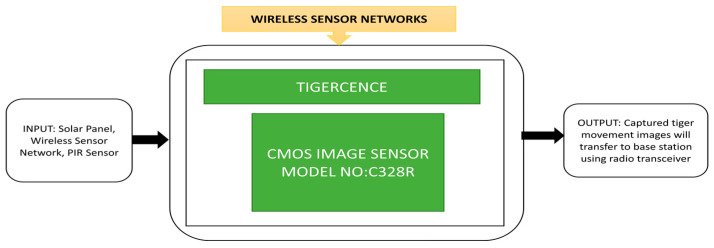
TigerCENSE.

Bagree et al. [6] developed a wireless image sensor network to monitor a tiger’s movements called “TigerCENSE.” It monitors previously unseen movements remotely and can be accessible in some dangerous places to watch the tiger’s behavior. A passive infrared sensor will activate this camera, and an integrated CMOS image sensor will capture the images that will be stored in the storage device. To avoid any white flashes that could disturbs the animal’s movements, an infrared flash is used to illuminate the scene during nighttime. The stored images will be sent to a remote station through a radio transceiver and will be transfered to the server database using internet link nodes. A solar panel is used to charge wireless nodes’ batteries to prevent frequent physical visits for battery changes. Tigers are usually differentiated by the stripe patterns on their body. No two tigers will have similar patterns. These patterns can help the researchers tigerCENSE to know their origin, presence, activity cycles, size of home ranges and behavior. The camera node will provide the tiger’s path and capture the tiger’s images using the infrared flash during the night. The prototype of the WSN was used for wildlife monitoring in field trials, and its black box approach is explained in Figure 53. TigerCENSE is reliable, nonintrusive, portable, consumes less power, and can be used in environments where humans cannot enter.

**Figure 54:**
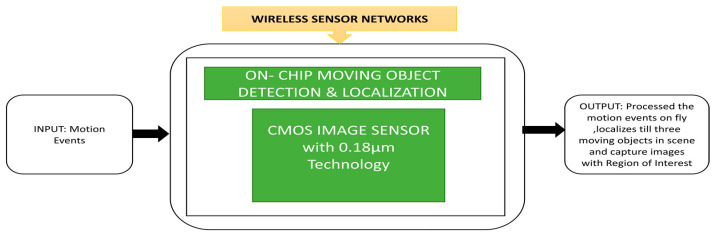
On chip moving object detection and localization using CMOS image sensor.

Zhao et al. [69] developed a moving object detection and localization CIS that can be integrated into wireless sensor nodes to perform robotic vision surveillance functions. This sensor will automatically change to the region of interest mode by obtaining the target object’s size and the position to capture the target image. The image sensor will capture grey level images during its normal intensity mode and capture the relevant temporal difference modes. A localization process will be applied to the temporal difference and automatically switch to the region of interest mode to capture the moving objects. Its black box approach is shown in Figure 54.

**Figure 55:**
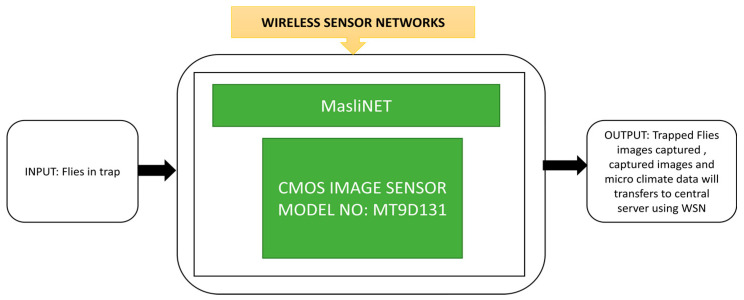
MasliNET-olive grove monitoring system using WSN.

Jelicic et al. [70] developed MasliNET, a multimodal environmental system that uses a wireless sensor network to monitor pests in olive groves. It also contains a self-energy harvesting unit to provide sufficient power for the system. MasliNET was deployed on a real olive farm. The image sensor can capture trapped flies’ images and send them to a server at a remote station. The system’s unique function is the lowest power consumption over a long period and the fact it works under the worst climatic conditions. Its black box approach is shown in Figure 55.

**Figure 56:**
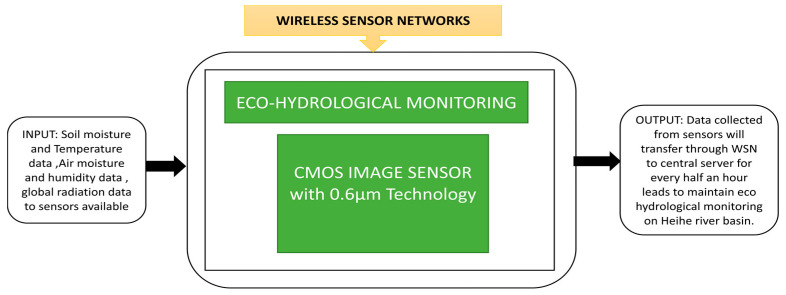
Eco-hydrological monitoring using WSN.

Luo et al. [71] developed a wireless sensor network to observe the Heihe river basin’s eco-hydrological process in which spatial dense parameters and material characteristics will be obtained. It is challenging to deploy networks in river basin areas due to the harsh climatic conditions and rough terrain. Suitable safety measures have been taken to deploy the network system at the basin area and gather the data to transfer via different repeaters to a remote station. Its operation with repeaters and its black box approach are clearly explained in Figure 56.

Controlling the pest population in agriculture is quite crucial in the case of forest and farm protection. Human labor is needed to perform frequent time-based trap surveys in agriculture fields, which consumes more time, labor, and is costly, especially for large fields or forest areas.

**Figure 57:**
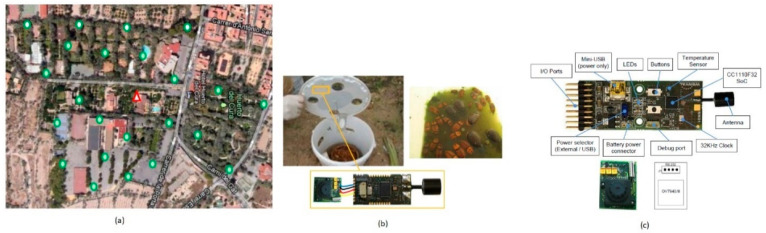
(**a**) Trap deployment aerial view; (**b**) Red Palm Weevil trap; (**c**) Image sensor used in trap [72].

Lopez et al. [72] proposed an image sensor-based autonomous monitoring system which captures the trap images and sends them with timing label to a remote station. This system can cover huge areas while consuming significantly little power. An efficient pest controlling technique is to keep these pest traps uniformly distributed over the specified target area to be controlled. This trap system is focused on controlling the red palm weevil pest, which severely affects palm fields across the world. Wireless image sensors are connected to traps placed in fields from a wireless image sensor network. An aerial view of trap deployment over a specific area is shown in Figure 57.

**Figure 58:**
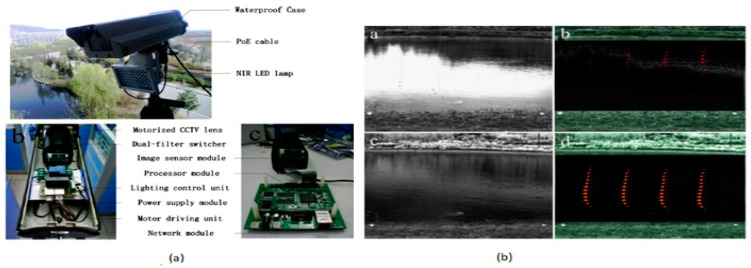
(**a**) Near Infra-Red (NIR) imaging camera with internal structure; (**b**) NIR captured images of river surface by applying LPSIV method in two different spectrum band with and without spatial high pass filtering. Adapted with permission from [73] Elsevier, 2013.

During natural disasters like floods, the water can be contaminated contaminated entirely with highly viscous mud and objects, which prevents the instruments from measuring the water flow. To understand the ecological and hydrological process of rivers set of data containing the water stage, hydrograph discharge and velocity distribution are needed. Large Scale Particle Image Velocimetry (LSPIV) is a powerful and proficient method to measure river surface velocity to analyze the river’s turbulence and flowing conditions in normal situations. Zhang et al. [73] developed a near-infrared imaging camera with internal preprocessing, image acquisition functions, as shown in Figure 58.

**Figure 59:**
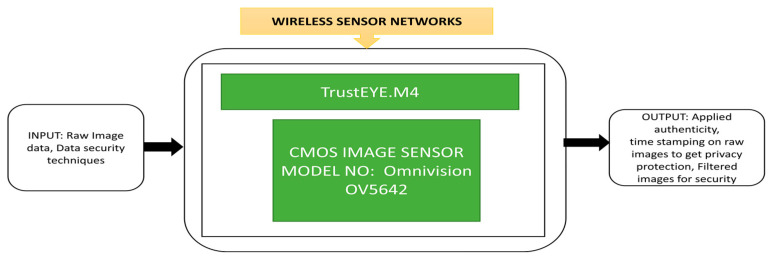
TrustEYE coupled with Raspberry Pi board having linux operating system.

The NIR camera is placed at the LPSIV site to capture and process the images to improve the contrast between the target objects and the background and increase the peak SNR. Using spatial high pass filtering, the noise and the river’s background will get suppressed efficiently, increasing the possibility of proper vectors in the immediate field flow. During the surveillance process in wireless sensor networks, cameras need to safely send the captured images to the remote station. Winkler et al. [74] proposed a unique feature in camera protection that involves security and privacy protection in an image sensing unit. Its black box approach is presented in Figure 59.

**Figure 60:**
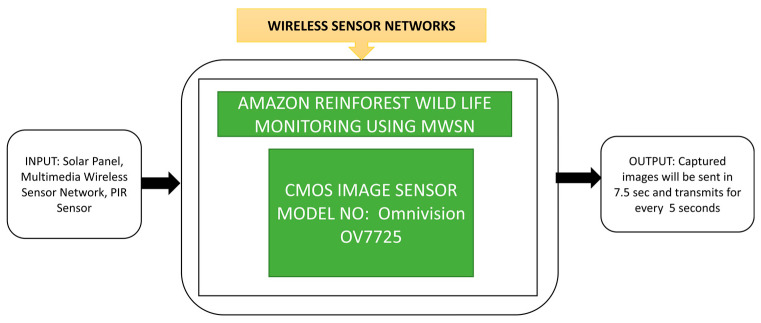
Amazon rainforest wildlife monitoring using multimedia wireless sensor networks (MWSN).

Multimedia wireless sensor networks (MWSN) utilization is increasing in various applications, including wildlife monitoring, environmental monitoring, etc. In wildlife monitoring, zoologists have started using trap cameras to capture animals’ images remotely without distributing them and trap cameras using MWSN to gather animals’ visual information and habitual behavior. Camacho et al. [75] designed a trap camera and implemented an MWSN to monitor wildlife in Peru’s Amazon rainforest. In this process, 25 trap cameras are installed and interconnected to form an MWSN at different forest locations. This monitoring experiment was conducted for six months by using 25 cameras in which only five cameras remained working at the end of the trial period. Its black box representation is presented in Figure 60.

**Figure 61:**
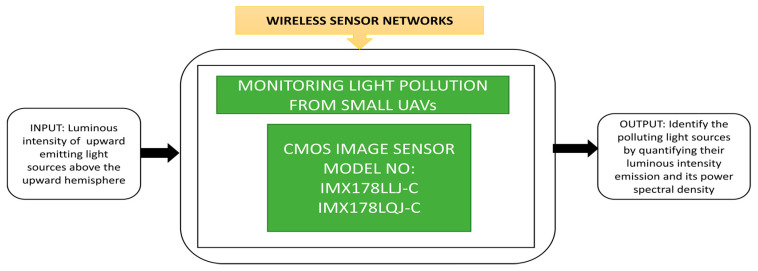
MINLU architecture for monitoring the light pollution from small UAV’s.

The author applied a cartoon filtering effect on the TrustEYE imaging sensing unit while capturing the images. This process will create privacy protection while detecting the region of interest. From the public transport and safety point of view, the outdoor lights along the roads, highways, parking lots, bicycle and pedestrian tracks must be switched on during nights and low light conditions. This in turn, causes light pollution, which must be calibrated. This calibration is needed, especially when Earth-observing satellites take images without blurring for better analysis. Fiorentini et al. [76] developed a sensor suite that can measure the intensity of luminance pollution of lights with a power spectral density function to detect the street lights’ various lamp technologies. At present, air balloons and drones are using in measuring light emission instead of satellites. An instrumental suite called “MINLU” can be used to monitor the light pollution generated by outdoor lighting with drones and air balloons. Its blackbox approach is shown in Figure 61.

The technical advancements in WSN applications is used in plant monitoring range from multispectral imaging to root phenotyping. A rhizotron imaging system is used to observe plant roots’ growth statistics at farms, parks or public places.

**Figure 62:**
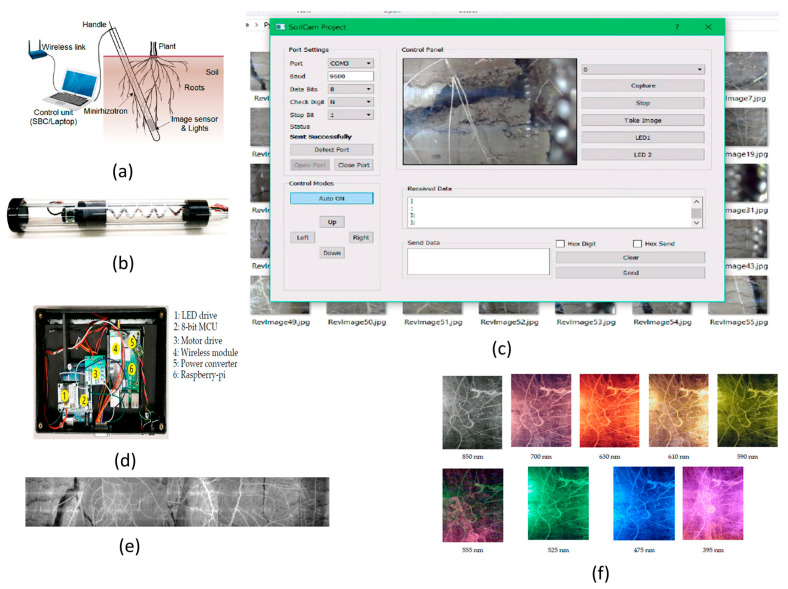
(**a**) Minirhizotron field experiment; (**b**) SoilCam; (**c**) Root and soil analyzer software; (**d**) Control box; (**e**) 360° image captured by SoilCam of a Canola plant root; (**f**) Multispectral images captured by SoilCam [77].

It is a nondestructive, underground, repetitive process system, which is costly to perform. To overcome this, Rahman et al. [77] designed the SoilCam: a mini rhizotron multispectral imaging system with fully automated functions to perform onsite recording, monitor the plant root growth process, and allow phenotyping research of plants, as shown in Figure 62.

## 4. Design Characteristics

### 4.1. CMOS Technology

**Figure 63:**
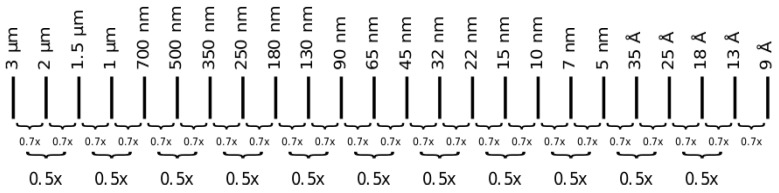
CMOS process technology variation [78].

According to Moore’s law, the transistor number on CMOS chips will double every couple of years, which means the integrated device’s speed will double. CMOS technology refers to the transistor gate length, which is reduced concerning the improvements and advancements occurring in the CMOS industry. Figure 63 presents the scaling of gate length to achieve double the density of transistors. The device’s total scaling will be done by scaling both contacted poly pitch and minimum metal pitch to 70% to achieve a 50% reduction in area.

### 4.2. Resolution

**Figure 64:**
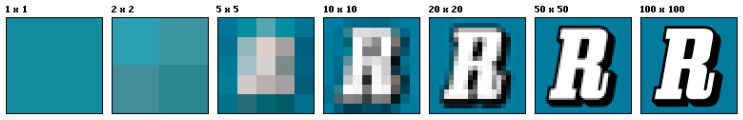
Various resolutions display [79].

This is a parameter that represents the number of pixels in an array. This pixel count will form a two-dimensional pixel array in which more number of pixels in an array can give more quality images, as shown in Figure 64.

### 4.3. Dynamic Range

The dynamic range is defined as the range of intensity of light in an image. The images produced by the image sensor with a high dynamic range are having light levels of low to high. The dynamic range’s mathematical representation is the ratio of maximum saturated pixel output level to the level of noise in the dark [8]:

(1) $$\mathrm{Dynamic}\;\mathrm{Range}\;=\;20\;\log\;\left(\frac{N_{sat}}{N_{dark}}\right)\left(\mathrm{dB}\right)$$

where *N_sat_* is the number of electrons collected by a pixel at saturation level, and this saturation level can be found by full well capacity. *N_dark_* is the number of electrons without illumination at the noise level.

### 4.4. Frame Rate

It is defined as the exposure time of the active pixel sensor and the frame readout time of the CMOS image sensor. If the exposure time decreases, then the frame rate increases, which means that the frame rate is inversely proportional to the exposure time. The units of the frame rate are frames per second (‘fps’) [8].

### 4.5. Signal to Noise Ratio (SNR)

SNR represents the pixel’s fidelity in the CMOS image sensor, which is defined as a ratio of input signal power to the referred noise power at the input [8]:

(2) $$\mathrm{SNR}\;=\;10\;\log_{10}\left(\frac{(i_{ph}t_{int})2}{q\left(i_{ph}+i_{dc}\right)t_{int}+\sigma_{read}2}\right)\;\mathrm{dB}$$

where *i_ph_* is the photocurrent, *i_dc_* is the dark current *t_int_* is the integration time $\sigma_{read}$ is the read noise.

**Figure 65:**
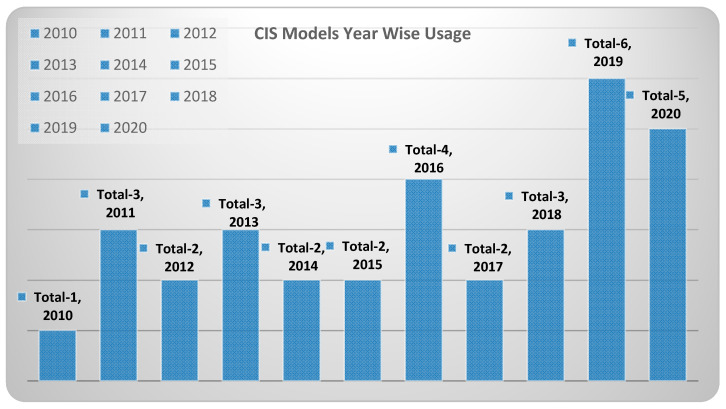
Year wise usage of CIS models according to survey data, where x-axis represents years and y axis represents number of CIS models.

The CIS models are nothing but camera modules used as imagers in surveillance applications discussed field-wise in this paper. The design characteristics play a key role in choosing an appropriate CIS model for a particular surveillance application in fields like the military and space, etc. The mostly used CIS models in surveillance systems are the OV7670, OV7725, OV2640, OV2710, OV9653, and OV9655 manufactured by Omnivision (Santa Clara, CA, USA), the MT9V125, MT9V131 by ON Semiconductor (Phoenix, AZ, USA), the MC1362 of Mikotron GmbH (Unterschleissheim, Germany), and the CMV4000, CMV20000 of AMS (Plano, TX, USA). Hence, we tabulated CIS models’ design characteristics termed as camera modules used in applications related to all the fields covered in our literature survey as shown in Table 1 and field-wise mapping of the CIS model, representing the CIS models used in each field as shown in Table 2. We have also tabulated the usage of CMOS image sensor models in applications by year in according with survey data having field-wise applications shown in Figure 65.

A lot of research and technological advancements are being made in surveillance systems. In security and biometric applications, gait recognition [80,81,82,83] has been introduced in surveillance systems to recognize the person by capturing his gestures while walking. This gait recognition technology can be operated from remote places using a server and also it can be applied to camera systems having low resolution to high resolution. The parameters like face and fingerprints datasets are not necessary to perform this gait recognition to identify people.

## 5. Discussion

### 5.1. Camera Models

From the data collected in our literature survey, we have mapped the CIS models incorporated in surveillance systems for various fields shown in Table 2. The remarkable models among them are EECAM for MARS 2020 mission, CUbE for driverless transportation of employers inside the industrial environment.

### 5.2. Future Directions

CIS has been used in many applications. It is technically improving very rapidly and being in more application areas. Quanta image sensors have evolved and have been developing gradually to overcome existing disadvantages compared to CCD. Due to the COVID outbreak, contactless technology is of interest in all applications, especially image capture, surveillance, money transactions like IRIS scanners and face recognition during the pandemic. New improvements were made in the medical field, and new algorithms are being implemented, such as face mask recognition in classrooms, especially for kids in school and playing zones.

## 6. Conclusions

Charge-coupled devices (CCD) played a vital role in many applications until CMOS image sensors came into existence. However, CIS still has some shortcomings to replacing CCDs in essential fields like the medical field and space, etc. To overcome CISs’ shortcoming, s various technological advancements have been introduced during the last decade and have made CIS a leading good competitor to CCD in the present market. CIS is highly in demand in all reputed cameras and high spectral imaging applications due to its low manufacturing cost and size.

## Figures and Tables

**Table 1:** Design Characteristics of CMOS image sensors.

S. No.	Year	Technology	Camera Module	Resolution	SNR (dB)	Frame Rate (fps)	Dynamic Range (dB)	Application Name/Target	Field
1	2009	0.35 µm	N/A	64 × 64	N/A	10	N/A	Built in lane Detection	Automotive
2	2011	0.18 µm	N/A	128 × 256	51	60	98	Night Vision Systems	Automotive
3	2012	N/A	MT9 V125	720 × 480	39	30	70	Fish-Eye Automotive Camera	Automotive
4	2013	0.18 µm	N/A	642 × 480	N/A	30	N/A	Optical Wireless Communication System	Automotive
5	2013	0.13 µm	N/A	768 × 576	45	N/A	70	On-Screen-Display (OSD)	Automotive
6	2014	0.18 µm	N/A	642 × 480	N/A	60	N/A	Visible Light Communication	Automotive
7	2015	N/A	GUPPY-F036 C	752 × 480	N/A	64	N/A	Traffic light Detection	Automotive
8	2015	0.35 µm	N/A	64 × 32	N/A	100	110	Three Dimensional Vision	Automotive
9	2018	0.11 µm	N/A	1280 × 1024	N/A	30	N/A	Non Contact Heart rate Detection	Automotive
10	2018	N/A	OV7725	640 × 480	50	60	60	Intelligent Car Path Tracking	Automotive
11	2011	N/A	OV6620	356 × 292	>48	60	>72	Nilaparvata Lugens Monitoring System	IoT
12	2011	N/A	OV7640	640 × 480	46	30	62	Crop Monitoring System	IoT
13	2011	N/A	Hercules Webcam	1280 × 960	N/A	30	N/A	Vine Yard Monitoring	IoT
14	2013	N/A	VBM40	1280 × 960	N/A	30	N/A	Human Monitoring System in Sea Transportation	IoT
15	2013	N/A	OV9655	1280 × 1024	N/A	15	N/A	Smart Camera Networks (SCN)	IoT
16	2016	0.18 µm	N/A	64 × 64	N/A	N/A	96.7	Smart Image Sensor with Multi Point Tracking (MPT)	IoT
17	2017	N/A	N/A	N/A	N/A	N/A	N/A	Early Flood Detection & Control Monitoring	IoT
18	2018	N/A	OV7670	640 × 480	40	30	52	Precision Agriculture System Design	IoT
19	2019	N/A	OV2640	1600 × 1200	40	15	50	SMART HOME	IoT
20	2019	N/A	SF3324-101	1928 × 1208	N/A	N/A	N/A	CUbE	IoT
21	2009	N/A	Quickcam Pro 9000	1600 × 1200	N/A	30	N/A	Privacy preserving sensor for Person Detection	ISS
22	2010	0.18 µm	N/A	64 × 64	N/A	30	N/A	surveillance in low crowded environments	ISS
23	2015	N/A	ucam-II	128 × 128	44.2	N/A	51	Visual surveillance and intrusion detection	ISS
24	2017	0.18 µm	N/A	176 × 144	47	14	61.8	Multi Resolution Mode	ISS
25	2018	0.09 µm	N/A	2560 × 1536	N/A	60	67	Moving Object Detection With Pre-defined Areas	ISS
26	2019	N/A	ZTE Nubia UINX511 J	5344 × 3000	N/A	120	N/A	Classroom Emotion with Cloud-Based Facial Recognizer	ISS
27	2019	N/A	DJI PHANTOM 3 PRO	4000 × 3000	N/A	N/A	N/A	vehicle Stacking Estimation	ISS
28	2020	N/A	OV2710-1 E	1920 × 1080	40	30	69	Nuclear Radiation Detection	ISS
29	2020	N/A	GS3-U3-23 S6 C-C	1920 × 1200	N/A	162	N/A	Contact less Neonatal Pulse Rate Sensing	ISS
30	2020	N/A	OV9653	1300 × 1028	40	15 to 120	62	HODET	ISS
31	2012	0.18 µm	N/A	368 × 368	N/A	10	49.2	Autonomous Micro Digital Sun Sensor	Space
32	2013	0.35 µm	N/A	256 × 256	N/A	N/A	N/A	Lightning Detection and Imaging	Space
33	2013	0.18 µm	N/A	320 × 128	N/A	N/A	126	STAR Tracking	Space
34	2016	N/A	CMV20000	5120 × 3840	41.8	0.45	66	MARS 2020 Mission: EECAM	Space
35	2017	N/A	MT9 M001 C12 STM	1280 × 1024	45	30	68.2	Cube SAT Remote Sensing Imagers	Space
36	2018	N/A	CMV4000	2048 × 2048	N/A	180	60	Cloud Monitoring Camera (CMC) System for Imaging Satellites	Space
37	2019	0.11 µm	N/A	3000 × 3000	45	N/A	72.4	Radiation Tolerant Sensor	Space
38	2019	N/A	IMX 264	2464 × 2056	N/A	60	N/A	Nanospacecraft Asteroid Flybys	Space
39	2019	N/A	OV9630	1280 × 1024	54	15	60	Mezn Sat for monitoring Green House Gases	Space
40	2020	N/A	CIS2521 F	2560 × 2160	N/A	100	>86	ASTERIA-A Space Telescope	Space
41	2010	N/A	OV9653	1300 × 1028	40	30	62	Wireless Aerial Image System	Millitary
42	2013	N/A	OV7725	640 × 480	50	60	60	IPASS	Millitary
43	2014	N/A	N/A	640 × 512	N/A	N/A	N/A	Banpil Camera	Millitary
44	2016	N/A	MPT 50	640 × 512	N/A	N/A	N/A	MWIR Detector for MissileApplications	Millitary
45	2016	N/A	PHOTRON SA4	1024 × 1024	80	3600	N/A	IN-SITU High velocity Rifle Bullets	Millitary
46	2016	N/A	OV7670	640 × 480	46	15	52	Wireless Vision sensor	Millitary
47	2017	N/A	Flea3	4000 × 3000	N/A	15	66.46	CARMA	Millitary
48	2018	N/A	ESN-0510	640 × 480	N/A	30	N/A	Sticky Bomb Detection	Millitary
49	2019	0.18 µm	N/A	64 × 64	N/A	200 k	N/A	Gun Muzzle Flash Detection System	Millitary
50	2020	N/A	MC1362	1280 × 1024	N/A	200 hz	90	Critical Part Detection of Reconnaissance Balloon	Millitary
51	2010	N/A	C328 R	640 × 480	N/A	N/A	N/A	Tigercense	WSN
52	2011	0.18 µm	N/A	64 × 64	N/A	100	N/A	On Chip Moving object Detection & Localization	WSN
53	2011	N/A	MT9 D131	1600 × 1200	42.3	15	71	MasliNET	WSN
54	2012	0.6 µm	N/A	384 × 288	N/A	N/A	N/A	Eco-Hydrological Monitoring	WSN
55	2012	N/A	C328-7640	640 × 480	46	30	62	Monitoring Pest Insect Traps	WSN
56	2013	N/A	MT9 M001	1280 × 1024	>45	30	>62	River Surface Target Enhancement	WSN
57	2014	N/A	OV5642	2592 × 1944	50	15	40	TrustEYE.M4	WSN
58	2014	N/A	OV7725	640 × 480	50	60	60	Wild life Inventory	WSN
59	2019	N/A	IMX178 LLJ-C	3088 × 2064	N/A	60	N/A	Monitoring light pollution from small UAVs	WSN
60	2019	N/A	ELP-USBFHD04 H-L170	1920 × 1080	39	30	72.4	SoilCam	WSN

**Table 2:** Field wise mapping of CMOS image sensor model.

*CMOS Image Sensor Model*	*Automotive*	*IoT*	*ISS*	*Military*	*Space*	*WSN*
***Aptina MT9 M001 C12 STM***					1	
***Cube SAT Remote Sensing Imagers***						
***C328-7640***						1
***Monitoring Pest Insect Traps***						
***CIS2521 F***					1	
***ASTERIA-A Space Telescope***						
***CMV20000***					1	
***MARS 2020 Mission: EECAM***						
***CMV4000***					1	
***Cloud Monitoring Camera (CMC) System for Imaging Satellites***						
***ELP-USBFHD04 H-L170***						1
***Soil Cam***						
***Flea3***				1		
***CARMA***						
***Grasshopper 3 GS3-U3-23 S6 C-C***			1			
***Contact less Neonatal Pulse Rate Sensing***						
***GUPPY-F036 C***	1					
***Traffic light Detection***						
***IMX 264***					1	
***Nano spacecraft Asteroid Flybys***						
***IMX178 LLJ-C IMX178 LQJ-C***						1
***Monitoring light pollution from small UAVs***						
***MC1362***				1		
***Critical Part Detection of Reconnaissance Balloon***						
***MPT 50***				1		
***MWIR Detector for Missile Applications***						
***MT9 D131***						1
***MasliNET***						
***MT9 M001***						1
***River Surface Target Enhancement***						
***MT9 V125***	1					
***Fish-Eye Automotive Camera***						
***OV2640***		1				
***SMART HOME***						
***OV2710-1 E***			1			
***Nuclear Radiation Detection***						
***OV5642***						1
***TrustEYE.M4***						
***OV6620***		1				
***Nilaparvata Lugens Monitoring System***						
***OV7640***		1				
***Crop Monitoring System***						
***OV7670***		1		1		
***Precision Agriculture***						
***Wireless Vision sensor***				1		
***OV7725***	1			1		1
***Intelligent Car Path Tracking***						
***IPASS***						
***Wild life Inventory***						1
***OV9630***					1	
***Mezn Sat for monitoring Green House Gases***						
***OV9653***			1	1		
***HODET***						
***Wireless Aerial Image System***				1		
***OV9655***		1				
***Smart Camera Networks (SCN)***						
***PHOTRON SA4***				1		
***IN-SITU High velocity Rifle Bullets***						
***Sekonix Camera SF3324-101***		1				
***CUbE***						
***ucam-II***			1			
***Visual surveillance and intrusion detection***						
***Grand Total (33)***	3	6	4	7	6	7

## Data Availability

The data used in this review is from published primary studies, which are available in the public domain.

## Abbreviations

The following abbreviations are used in this manuscript:

CIS	CMOS Image Sensor
DR	Dynamic Range
SNR	Signal to Noise Ratio
FPS	Frames Per Second
dB	Decibel
mm	Millimeter
µm	Micrometer
v/lux.sec	Volts per luminance. second
CCD	Charge Coupled Devices
CMOS	Complementary Metal Oxide Semiconductor
IoT	Internet of Things
ISS	Intelligent Surveillance Systems
WSN	Wireless Sensor Networks
BSI	Back Side Illumination
WDR	Wide Dynamic Range

## Appendix A

**Table A1:** Taxonomy of the current literature.

S. No.	Year	Camera Module	Design Specifications					Application Name/Target	Field	Reference
			Technology	Resolution	SNR	Frame Rate	Dynamic Range			
1	2020	OV2710-1 E	--	YES	YES	YES	YES	Nuclear Radiation Detection	ISS	Zhangfa Yan et al. [25]
2	2020	GS3-U3-23 S6 C-C	--	YES	No	YES	No	Contact less Neonatal Pulse Rate Sensing	ISS	M. Paul et al. [26]
3	2020	OV9653	--	YES	YES	YES	YES	HODET	ISS	Joseph st. Cyr et al. [1]
4	2020	CIS2521 F	--	YES	No	YES	YES	ASTERIA-A Space Telescope	Space	Mary Knapp et al. [45]
5	2020	MC1362	--	YES	No	YES	YES	Critical Part Detection of Reconnaissance Balloon	Millitary	Hanyu Hong et al. [57]
6	2019	OV2640	--	YES	YES	YES	YES	SMART HOME	IoT	Vivek Raj et al. [34]
7	2019	Sekonix SF3324-101	--	YES	No	No	No	CUbE	IoT	A. Hartmannsgruber et al. [35]
8	2019	ZTE Nubia UINX511 J	--	YES	No	YES	No	Classroom Emotion with Cloud-Based Facial Recognizer	ISS	C. Boonroungrut et al. [23]
9	2019	DJI PHANTOM 3 PRO	--	YES	No	No	No	Vehicle Stacking Estimation	ISS	Brain S. Freeman et al. [24]
10	2019	--	0.11 µm	YES	YES	No	YES	Radiation Tolerant Sensor	Space	Woo-Tae Kim et al. [42]
11	2019	IMX 264	--	YES	No	YES		Nanospacecraft Asteroid Flybys	Space	Mihkel Pajusalu et al. [43]
12	2019	OV9630	--	YES	YES	YES	YES	Mezn Sat for monitoring Green House Gases	Space	Halim Jallad et al. [4]
13	2019	--	0.18 µm	YES	No	YES	No	Gun Muzzle Flash Detection System	Millitary	Alex Katz et al. [56]
14	2019	IMX178 LLJ,QJ-C	--	YES	No	YES	No	Monitoring light pollution from small UAVs	WSN	Fiorentin et al. [76]
15	2019	ELP-USBFHD04 H-L170	--	YES	YES	YES	YES	SoilCam	WSN	Gazi Rahman et al. [77]
16	2018	--	0.11 µm	YES	No	YES	No	Non Contact Heart rate Detection	Automotive	Chen Cao et al. [60]
17	2018	OV7725	--	YES	YES	YES	YES	Intelligent Car Path Tracking	Automotive	Zixin Mu et al. [68]
18	2018	OV7670	--	YES	YES	YES	YES	Precision Agriculture System Design	IoT	Arun M.Patokar et al. [33]
19	2018	--	0.09 µm	YES	No	YES	YES	Moving Object Detection With Pre-defined Areas	ISS	Oichi Kumagai et al. [22]
20	2018	CMV4000	--	YES	No	YES	YES	Cloud Monitoring Camera (CMC) System for Imaging Satellites	Space	Alpesh vala et al. [41]
21	2018	ESN-0510	--	YES	No	YES	No	Sticky Bomb Detection	Millitary	Raed Majeed et al. [55]
22	2017	--	--	No	No	No	No	Early Flood Detection & Control Monitoring	IoT	T M Thekkil et al. [32]
23	2017	--	0.18 µm	YES	YES	YES	YES	Multi Resolution Mode	ISS	Daehyeok Kim et al. [21]
24	2017	MT9 M001 C12 STM	--	YES	YES	YES	YES	Cube SAT Remote Sensing Imagers	Space	Dee W. Pack et al. [40]
25	2017	Flea3	--	YES	No	YES	YES	CARMA	Millitary	Shannon Johnson et al. [53]
26	2016	--	0.18 µm	YES	No	No	YES	Smart Image Sensor with Multi Point Tracking (MPT)	IoT	Chin Yin et al. [31]
27	2016	CMV20000	--	YES	YES	YES	YES	MARS 2020 Mission: EECAM	Space	Mckinney et al. [39]
28	2016	MPT 50	--	YES	No	No	No	MWIR Detector for Missile Applications	Millitary	Ulas Kurum et al. [50]
29	2016	PHOTRON SA4	--	YES	YES	YES	No	IN-SITU High velocity Rifle Bullets	Millitary	J.D’Aries et al. [51]
30	2016	OV7670	--	YES	YES	YES	YES	Wireless Vision sensor	Millitary	Parivesh Pandey et al. [52]
31	2015	GUPPY-F036 C	--	YES	No	YES	No	Traffic light Detection	Automotive	Moises Diaz et al. [67]
32	2015	--	0.35 µm	YES	No	YES	YES	Three Dimensional Vision	Automotive	Danilo Bronzi et al. [64]
33	2015	ucam-II	--	YES	YES	No	YES	Visual surveillance and intrusion detection	ISS	Congduc Pham et al. [12]
34	2014	--	0.18 µm	YES	No	YES	No	Visible Light Communication	Automotive	Takaya Yamazato et al. [62]
35	2014	--	--	YES	No	No	No	Banpil Camera	Millitary	Patrick Odour et al. [49]
36	2014	OV5642	--	YES	YES	YES	YES	TrustEYE.M4	WSN	Thomas Winkler et al. [74]
37	2014	OV7725	--	YES	YES	YES	YES	Wild life Inventory	WSN	Luis Camacho et al. [75]
38	2013	--	0.18 µm	YES	No	YES	No	Optical Wireless Communication System	Automotive	Isamu Takai et al. [63]
39	2013	--	0.13 µm	YES	YES	No	YES	On-Screen-Display (OSD)	Automotive	Sheng Zhang et al. [59]
40	2013	VBM40	--	YES	No	YES	No	Human Monitoring System in Sea Transportation	IoT	Masakazu Arima et al. [3]
41	2013	OV9655	--	YES	No	YES	No	Smart Camera Networks (SCN)	IoT	Phoebus Chen et al. [30]
42	2013	--	0.35 µm	YES	No	No	No	Lightning Detection and Imaging	Space	Sebastien Rolando et al. [37]
43	2013	--	0.18 µm	YES	No	No	YES	STAR Tracking	Space	Xinyuan Qian et al. [38]
44	2013	OV7725	--	YES	YES	YES	YES	IPASS	Millitary	Adam Blumenau et al. [5]
45	2013	MT9 M001	--	YES	YES	YES	YES	River Surface Target Enhancement	WSN	Zhen Zhang et al. [73]
46	2012	MT9 V125	--	YES	YES	YES	YES	Fish-Eye Automotive Camera	Automotive	Mauro Turturici et al. [2]
47	2012	--	0.18 µm	YES	No	YES	YES	Autonomous Micro Digital Sun Sensor	Space	Ning Xie et al. [36]
48	2012		0.6 µm	YES	No	No	No	Eco-Hydrological Monitoring	WSN	L. Luo et al. [71]
49	2012	C328-7640	--	YES	YES	YES	YES	Monitoring Pest Insect Traps	WSN	Otoniel Lopez et al. [72]
50	2011	--	0.18 µm	YES	YES	YES	YES	Night Vision Systems	Automotive	Arthur Spivak et al. [66]
51	2011	OV6620	--	YES	YES	YES	YES	Nilaparvata Lugens Monitoring System	IoT	Ken Cai et al. [27]
52	2011	OV7640	--	YES	YES	YES	YES	Crop Monitoring System	IoT	Zhao Liqiag et al. [28]
53	2011	Hercules Webcam	--	YES	No	YES	No	Vine Yard Monitoring	IoT	Jaime Lloret et al. [29]
54	2011	--	0.18 µm	YES	No	YES	No	On Chip Moving object Detection & Localization	WSN	Bo Zhao et al. [69]
55	2011	MT9 D131	--	YES	YES	YES	YES	MasliNET	WSN	Vana Jelicic et al. [70]
56	2010		0.18 µm	YES	No	YES	No	Surveillance in low crowded environments	ISS	Mehdi Habibi et al. [11]
57	2010	OV9653	--	YES	YES	YES	YES	Wireless Aerial Image System	Millitary	Li Zhang et al. [48]
58	2010	C328 R	--	YES	No	No	No	Tigercense	WSN	Ravi Bagree et al. [6]
59	2009	--	0.35 µm	YES	No	YES	No	Built in lane Detection	Automotive	Pei-yung Hsiao et al. [58]
60	2009	Logitech Pro 9000	--	YES	No	YES	No	Privacy preserving sensor for Person Detection	ISS	Shota Nakashima et al. [10]

**Table A2:** Parametric data of CMOS image sensors used in surveillance system applications.

Year	Technology	Camera Module	Pixel Size (µm)	Resolution	Pixel Pitch (µm)	Area	Power (w/mw)	Dark Current (mv/s)	SNR (dB)	Conversion Gain (µV/e-)	Sensitivity (V/lux-s)	Frame Rate (fps)	Dynamic Range (dB)	Field	Application Name/Target
2009	N/A	Logitech Quickcam Pro 9000	N/A	1600 × 1200	N/A	N/A	N/A	N/A	N/A	N/A	N/A	30 fps	N/A	ISS	Privacy reserving sensor for Person Detection
2010	0.18 µm	N/A	7.5 × 7.5	64 × 64	7.5	1 mm × 1 mm	0.5 mw	N/A	N/A	N/A	N/A	30 fps	N/A	ISS	Surveillance in low crowded areas
2015	N/A	ucam-II	5.5 × 5.5	128 × 128	5.5	27.5 mm × 32.5 mm	N/A	25.2	44.2	N/A	2.93 V/lux-s	N/A	51	ISS	Visual surveillance and intrusion detection
2017	0.18 µm	N/A	4.4 × 4.4	176 × 144	4.4	2.35 mm × 2.35 mm	10 mw-HDR	N/A	47 dB-HDR	N/A	--	14 fps	61.8	ISS	Multi Resolution Mode
2018	0.09 µm	N/A	1.5 × 1.5	2560 × 1536	1.5	4.48 mm × 4.48 mm	95 mw	N/A	N/A	55.8	8033 e-/lux-s	60 fps	67	ISS	Moving Object Detection With Pre-defined Areas
2019	N/A	ZTE Nubia UINX511 J	N/A	5344 × 3000	N/A	6.828 mm × 6.828 mm	N/A	N/A	N/A	N/A	N/A	120 fps	N/A	ISS	Classroom Emotion with Cloud-Based Facial Recognizer
2019	N/A	DJI PHANTOM 3 PRO	N/A	4000 × 3000	N/A	N/A	N/A	N/A	N/A	N/A	N/A	N/A	N/A	ISS	vehicle Stacking Estimation
2020	N/A	OV2710-1 E	3 × 3	1920 × 1080	3	5886 µm × 3276 µm	350 mW	20	40	N/A	3.7	30 fps	69	ISS	Nuclear Radiation Detection
2020	N/A	GS3-U3-23 S6 C-C	5.86 × 5.86	1920 × 1200	5.86	N/A	N/A	N/A	N/A	N/A	N/A	162 fps	N/A	ISS	Contact less Neonatal Pulse Rate Sensing
2020	N/A	OV9653	3.18 × 3.18	1300 × 1028	3.18	4.13 mm × 3.28 mm	50 mw	30	40	N/A	0.9	15–120 fps	62	ISS	HODET
2011	N/A	OV6620	9.0 × 8.2	356 × 292	9.0	3.1 mm × 2.5 mm	<80 mw	<0.2 nA /cm^2^	>48	N/A	N/A	60 fps	>72	IoT	Nilaparvata Lugens Monitoring System
2011	N/A	OV7640	5.6 × 5.6	640 × 480	5.6	3.6 mm × 2.7 mm	40 mw	30	46	N/A	3.0-Black /Color-1.12	30 fps	62	IoT	Crop Monitoring System
2011	N/A	Hercules Webcam	N/A	1280 × 960	N/A	N/A	N/A	N/A	N/A	N/A	N/A	30 fps	N/A	IoT	Vine Yard Monitoring
2013	N/A	VBM40	N/A	1280 × 960	N/A	132 mm × 152 mm	N/A	N/A	N/A	N/A	0.6 V/lux	30 fps	N/A	IoT	Human Monitoring System in Sea Transportation
2013	N/A	OV9655	3.18 × 3.18	1280 × 1024	3.18	5145 µm × 6145 µm	90 mw	N/A	N/A	N/A	N/A	15 fps	N/A	IoT	Smart Camera Networks (SCN)
2016	0.18 µm	N/A	20 × 20	64 × 64	20	N/A	N/A	N/A	N/A	N/A	N/A	N/A	96.7	IoT	Smart Image Sensor with Multi Point Tracking (MPT)
2017	N/A	N/A	N/A	N/A	N/A	N/A	N/A	N/A	N/A	N/A	N/A	N/A	N/A	IoT	Early Flood Detection & Control Monitoring
2018	N/A	OV7670	3.6 × 3.6	640 × 480	3.6	2.36 mm × 1.76 mm	N/A	12 mv/s	40	N/A	1.1 V/lux−sec	30 fps	52	IoT	Precision Agriculture System Design
2019	N/A	OV2640	2.2 × 2.2	1600 × 1200	2.2	3590 µm × 2684 µm	125 mW	15	40	N/A	0.6	15 fps	50	IoT	SMART HOME
2019	N/A	Sekonix SF3324-101	3 × 3	1928 × 1208	3	26 mm × 26 mm	N/A	N/A	N/A	N/A	N/A	N/A	N/A	IoT	CUbE
2012	0.18 µm	N/A	N/A	368 × 368	N/A	5 mm × 5 mm	21.34 mw@ Acquisition 21.39 mW @ Tracking	N/A	N/A	N/A	N/A	10 fps	49.2 dB	Space	Autonomous Micro Digital Sun Sensor
2013	0.35 µm	N/A	60 × 60	256 × 256	60	17.8 mm × 17.8 mm	N/A	N/A	N/A	5.7	N/A	N/A	N/A	Space	Lightning Detection and Imaging
2013	0.18 µm	N/A	5 × 5	320 × 128	5	2.5 mm × 2.5 mm	247 mW	1537 fA	N/A	N/A	0.25	N/A	126 dB	Space	STAR Tracking
2016	N/A	CMV20000	6.4 × 6.4	5120 × 3840	6.4	32.77 mm × 24.58 mm	<3 w	125 e-/s	41.8 dB	0.25	N/A	0.45 fps	66 dB	Space	MARS 2020 Mission: EECAM
2017	N/A	MT9 M001 C12 STM	5.2 × 5.2	1280 × 1024	5.2	6.6 mm × 5.32 mm	363 mW	N/A	45 dB	N/A	2.1	30 fps	68.2 dB	Space	Cube SAT Remote Sensing Imagers
2018	N/A	CMV4000	5.5 × 5.5	2048 × 2048	5.5	N/A	650 mw	125 e-/s	N/A	0.075 LSB/e-	5.56	180 fps	60 dB	Space	Cloud Monitoring Camera (CMC) System for Imaging Satellites
2019	0.11 µm	N/A	6.5 × 6.5	3000 × 3000	6.5	22 mm × 22 mm	N/A	N/A	45 dB	8.55	N/A	N/A	72.4 dB	Space	Radiation Tolerant Sensor
2019	N/A	IMX 264	3.45 × 3.45	2464 × 2056	3.45	N/A	N/A	N/A	N/A	N/A	0.915	60 fps	N/A	Space	Nano spacecraft Asteroid Flybys
2019	N/A	OV9630	4.2 × 4.2	1280 × 1024	4.2	5.4 mm × 4.3 mm	150 mW	28 mv	54 dB	N/A	1	15 fps	60 dB	Space	Mezn Sat for monitoring Green House Gases
2020	N/A	CIS2521 F	6.5 × 6.5	2560 × 2160	6.5	N/A	N/A	35 e-/s	N/A	N/A	N/A	100 fps	>86 dB	Space	ASTERIA-A Space Telescope
2010	N/A	OV9653	3.18 × 3.18	1300 × 1028	3.18	4.13 mm × 3.28 mm	50 mw	30	40 dB	N/A	0.9	30 fps	62 dB	Military	Wireless Aerial Image System
2013	N/A	OV7725	6.0 × 6.0	640 × 480	6	3984 µm × 2952 µm	120 MW	40 mV/s	50 dB	N/A	3	60 fps	60 dB	Military	IPASS
2014	N/A	N/A	N/A	640 × 512	N/A	9.6 mm × 7.7 mm	N/A	N/A	N/A	N/A	N/A	N/A	N/A	Military	Banpil Camera
2016	N/A	MPT 50	15 × 15	640 × 512	15	N/A	N/A	N/A	N/A	N/A	N/A	N/A	N/A	Military	MWIR Detector for Missile Applications
2016	N/A	PHOTRON SA4	20 × 20	1024 × 1024	20	160 mm × 153 mm	N/A	N/A	80 dB	N/A	N/A	3600 fps	N/A	Military	IN-SITU High velocity Rifle Bullets
2016	N/A	OV7670	3.6 × 3.6	640 × 480	3.6	2.36 mm × 1.76 mm	60 mW	12	46 dB	N/A	1.3	15 fps	52 dB	Military	Wireless Vision sensor
2017	N/A	Flea3	1.55 × 1.55	4000 × 3000	1.55	29 mm × 29 mm	N/A	N/A	N/A	N/A	N/A	15 fps	66.46 dB	Military	CARMA
2018	N/A	ESN-0510	N/A	640 × 480	N/A	N/A	N/A	N/A	N/A	N/A	N/A	30 fps	N/A	Military	Sticky Bomb Detection
2019	0.18 µm	N/A	54 × 54	64 × 64	54	5 mm × 5.5 mm	N/A	N/A	N/A	N/A	N/A	200 kfps	N/A	Military	Gun Muzzle Flash Detection System
2020	N/A	MC1362	14 × 14	1280 × 1024	14	17.92 mm × 14.34 mm	N/A	0.2	N/A	N/A	25	200 hz	90 dB	Military	Critical Part Detection of Reconnaissance Balloon
2009	0.35 µm	N/A	13.1 × 22.05	64 × 64	N/A	2194.4 µm × 2389.8 µm	159.4 mw	N/A	N/A	N/A	N/A	10 fps	N/A	Automotive	Built in lane Detection
2011	0.18 µm	N/A	13.75 × 13.75	128 × 256	8.5	N/A	10 mw	0.1 fA	51 db	72	N/A	60 fps	98 dB	Automotive	Night Vision Systems
2012	N/A	MT9 V125	5.6 × 5.6	720 × 480	5.6	3.63 mm × 2.78 mm	320 mw	N/A	39 dB	N/A	N/A	30 fps	70 dB	Automotive	Fish-Eye Automotive Camera
2013	0.18 µm	N/A	7.5 × 7.5	642 × 480	7.5	7.5 mm × 8.0 mm	N/A	N/A	N/A	N/A	N/A	30 fps	N/A	Automotive	Optical Wireless Communication System
2013	0.13 µm	N/A	6.0 × 6.0	768 × 576	6	N/A	N/A	N/A	45 db	95	4.8 V/lux-sec	N/A	70 dB	Automotive	On-Screen-Display (OSD)
2014	0.18 µm	N/A	7.5 × 7.5	642 × 480	7.5	7.5 mm × 8.0 mm	N/A	N/A	N/A	N/A	N/A	60 fps	N/A	Automotive	Visible Light Communication
2015	N/A	GUPPY-F036 C	6.0 × 6.0	752 × 480	6	N/A	N/A	N/A	N/A	N/A	N/A	64 fps	N/A	Automotive	Traffic light Detection
2015	0.35 µm	N/A	150 × 150	64 × 32	150	N/A	4 w	N/A	N/A	N/A	N/A	100 fps	110 dB	Automotive	Three-Dimensional Vision
2018	0.11 µm	N/A	7.1 × 7.1	1280 × 1024	7.1	12.6 mm × 14.8 mm	N/A	N/A	N/A	99.2	134.8 ke-/lux-s	30 fps	N/A	Automotive	Non-Contact Heart Rate Detection
2018	N/A	OV7725	6.0 × 6.0	640 × 480	6	3984 µm × 2952 µm	120 mW	40 mv/s	50 dB	N/A	3.8 V/lux-sec	60 fps	60 dB	Automotive	Intelligent Car Path Tracking
2010	N/A	C328 R	N/A	640 × 480	2	20 mm × 28 mm	N/A	N/A	N/A	N/A	N/A	N/A	N/A	WSN	Tigercense
2011	0.18 µm	N/A	14 × 14	64 × 64	14	1.5 mm × 1.5 mm	0.4 mw	6.7 fA	N/A	N/A	0.11 V/lux-s	100 fps	N/A	WSN	On Chip Moving object Detection & localization
2011	N/A	MT9 D131	2.8 × 2.8	1600 × 1200	2.8	4.73 mm × 3.52 mm	348 mw	N/A	42.3 dB	N/A	1.0 V/lux-sec	15 fps	71 dB	WSN	MasliNET
2012	0.6 µm	N/A	24 × 24	384 × 288	24	11.5 mm × 7.7 mm	150 mW	N/A	N/A	N/A	N/A	N/A	N/A	WSN	Eco-Hydrological Monitoring
2012	N/A	C328-7640	5.6 × 5.6	640 × 480	5.6	3.6 mm × 2.7 mm	40 mw	30 mv/s	46 dB	N/A	3.0 V/lux-s --B&W, 1.12 V/Lux-S --Colour	30 fps	62 dB	WSN	Monitoring Pest Insect Traps
2013	N/A	MT9 M001	5.2 × 5.2	1280 × 1024	5.2	N/A	325 mw	20–30 e-s	>45 dB	N/A	1.8 v/lux-sec	30 fps	>62 dB	WSN	River Surface Target Enhancement
2014	N/A	OV5642	1.4 × 1.4	2592 × 1944	1.4	3673.6 µm × 2738.4 µm	N/A	N/A	50 dB	N/A	0.6 V/lux-sec	15 fps	40 dB	WSN	TrustEYE.M4
2014	N/A	OV7725	6 × 6	640 × 480	6	3984 µm × 2952 µm	120 mW	40 mv/s	50 dB	N/A	3.8 V/lux-sec	60 fps	60 dB	WSN	Wild life Inventory
2019	N/A	IMX178 LLJ-C IMX178 LQJ-C	2.4 × 2.4	3088 × 2064	2.4	8.92 mm × 8.92 mm	N/A	N/A	N/A	N/A	0.38, 0.425	60 fps	N/A	WSN	Monitoring light pollution from small UAVs
2019	N/A	ELP-USBFHD04 H-L170	2.2 × 2.2	1920 × 1080	2.2	32 mm × 32 mm	N/A	N/A	39 dB	N/A	1.9 v/lux-sec	30 fps	72.4 dB	WSN	SoilCam
